# Body size preferences for women and adolescent girls living in Africa: a mixed-methods systematic review

**DOI:** 10.1017/S1368980021000768

**Published:** 2022-03

**Authors:** Rebecca Pradeilles, Michelle Holdsworth, Oluwabukola Olaitan, Ana Irache, Hibbah A Osei-Kwasi, Christian B Ngandu, Emmanuel Cohen

**Affiliations:** 1 School of Sport, Exercise and Health Sciences (SSEHS), Loughborough University, Loughborough LE11 3TU, UK; 2 UMR MoISA (Montpellier Interdisciplinary Centre on Sustainable Agri-food Systems), (Univ Montpellier, CIRAD, CIHEAM-IAMM, INRAE, Institut Agro, IRD), Montpellier, France; 3 University Hospitals of Leicester NHS trust, Leicester, UK; 4 Warwick Medical School, University of Warwick, Coventry, UK; 5 Department of Geography, University of Sheffield, Sheffield, UK; 6 MRC/Wits Developmental Pathways for Health Research Unit, Department of Paediatrics, University of the Witwatersrand, Johannesburg, South Africa; 7 UMR CNRS-MNHN 7206 « Eco-anthropologie », Musée de l’Homme, Paris, France

**Keywords:** Body size preferences, Women, Adolescent girls, Africa, Review

## Abstract

**Objective:**

To synthesise evidence on body size preferences for females living in Africa and the factors influencing these.

**Design:**

Mixed-methods systematic review including searches on Medline, CINHAL, ASSIA, Web of Science and PsycINFO (PROSPERO CRD42015020509). A sequential-explanatory approach was used to integrate quantitative and qualitative findings.

**Setting:**

Urban and rural Africa.

**Participants:**

Studies of both sexes providing data on body size preferences for adolescent girls and women aged ≥10 years.

**Results:**

Seventy-three articles from twenty-one countries were included: fifty quantitative, fifteen qualitative and eight mixed methods. Most studies reported a preference for normal or overweight body sizes. Some studies of adolescent girls/young women indicated a preference for underweight. Factors influencing preferences for large(r) body sizes included: socio-demographic (e.g. education, rural residency), health-related (e.g. current BMI, pubertal status), psycho-social (e.g. avoiding HIV stigma) and socio-cultural factors (e.g. spouse’s preference, social standing, cultural norms). Factors influencing preferences for slim(mer) body sizes included: socio-demographic (e.g. higher socioeconomic status, urban residency, younger age), health-related (e.g. health knowledge, being nulliparous), psycho-social (e.g. appearance, body size perception as overweight/obese) and socio-cultural factors (e.g. peer pressure, media).

**Conclusions:**

Preference for overweight (not obese) body sizes among some African females means that interventions need to account for the array of factors that maintain these preferences. The widespread preference for normal weight is positive in public health terms, but the valorisation of underweight in adolescent girls/young women may lead to an increase in body dissatisfaction. Emphasis needs to be placed on education to prevent all forms of malnutrition.

The prevalence of overweight including obesity has increased rapidly amongst women in African countries, from 33·0 % in 2000 to 42·9 % in 2016^(1)^. North African and Southern Sub-Saharan African women experience the second and third highest prevalence of overweight and obesity of the twenty one global burden of disease regions^(2)^, which are higher than the global average^(3)^.

Current literature suggests that there are underlying social and cultural factors related to body image, such as a preference for larger body sizes, contributing to the increasing prevalence of obesity in African females^(4,5)^. Body size preference plays a role in healthy weight behaviours. It is defined as an individual’s perception of how acceptable their body is to themselves and society^(6)^. This includes body image dissatisfaction that may contribute to attempting to achieve an ideal body image leading to a desire for weight loss or weight gain^(7)^. Body dissatisfaction (for slimness) may be one of the key drivers of the obesity epidemic in African women and adolescent girls. A cultural preference for a heavier body size is thought to lead to greater body satisfaction of African women at larger body sizes^(8)^. In addition, body dissatisfaction might promote weight gain in slimmer women trying to fit in with cultural norms. For example, Moroccan Sahraouian women actively engage in fattening behaviours to attain the desirable body size within their community^(9)^. Fatness is a sign of femininity, fertility and being a nurturing mother. Women’s primary role in these societies is associated with motherhood, therefore, ‘being fat’ elevates females’ status by embodying their suitability for this role^(10,11)^. Historically, fatness was viewed as a sign of wealth, signifying excess resources in settings where food shortages were common and also demonstrating that women were well taken care of by their husbands^(11)^.

This preference for a larger body size in women can be explained by evolutionary benefits. Prior to the industrialisation of food production, food shortages were common in all societies; therefore, storing fat improved survival^(10,12)^. This was particularly important for women of childbearing age, meaning fatter women would be more successful in pregnancy and childbearing^(10,13)^. Notwithstanding, in certain African communities, weight gain during pregnancy is perceived negatively due to the fear of complications during delivery^(14–16)^. The belief that fatness represents an advantage for pregnancy and childbearing might persevere in some African societies due to the persistence of undernutrition secondary to poverty, particularly in rural areas^(17)^, potentially making fatness desirable, by setting one apart from the community and embodying excess in resource poor settings^(10)^. This is supported by the positive association between socio-economic status (SES) and obesity in low-income African countries^(18,19)^, whereas in high-income countries (HICs) the opposite is observed^(20)^. In African middle-income countries, the association between SES and obesity is negative for women^(20)^. Furthermore, Western body size preferences have shifted towards a slimmer body size, whilst the population’s mean BMI has increased, suggesting that body size preferences evolve to contradict societal norms^(21,22)^.

An aversion to ‘thinness’ may also exist in low-income African countries because of stigmatisation associated with disease, for example, HIV and tuberculosis (TB)^(23,24)^.

In addition to cultural factors contributing to body size preference, studies from HICs have reported that being overweight, media exposure and psychological factors contribute to higher body dissatisfaction^(6,25–28)^. Younger females are more likely to want to change their body size to fit emerging societal norms valuing thinness^(6)^. Therefore, access to ‘Western’ media in Africa might increase the value of slimmer bodies, particularly amongst younger African females. Adolescence signifies the onset of puberty, which increases awareness of body image directly and indirectly via a greater importance placed on peer perceptions^(29)^. Indeed, studies have reported an increased ‘drive for thinness’ in Black South African adolescents when compared with their White South African counterparts. This contradicts studies of fattening practices in African women^(9,30)^. This might be a result of transitioning cultural norms and generational differences.

In light of the increasing obesity epidemic in African women and the contradictory results reported from the studies discussed earlier^(8,30–32)^, this paper presents the results of a mixed methods systematic review, to assess body size preferences for African women and adolescent girls living in Africa and the factors influencing these preferences.

## Methods

### Review typology

A mixed methods systematic review was chosen as it combines quantitative and qualitative evidence^(33)^ generating a complete, and deeper understanding of women’s body size preferences and the factors influencing these (PROSPERO #: CRD42015020509).

### Inclusion and exclusion criteria

Inclusion criteria were based on the SPIDER tool (Sample, Phenomenon of Interest, Design, Evaluation and Research Type) (Table 1). Studies conducted in any African country among female adolescents (10–19 years) and women (≥18 years) were included. The review focused exclusively on Black African or Arab females. All studies that assessed preferred body size of African females (adolescent girls and women) using narrative and/or pictorial measures, and those that elicited factors influencing these preferences were included. Furthermore, studies assessing African males’ preferences for African females’ body size were included. All non-randomised quantitative, qualitative and mixed methods studies were eligible for inclusion.

**Table 1:** SPIDER breakdown of research question and search terms

Category	Sample (S)	Phenomenon of interest (PofI)	Design (D)	Evaluation (E)	Research type (R)
Research question	African females and males, aged 10 and above residing in Africa	Preferred body size; desire to gain or lose weight.	Non-randomised quantitative studies; qualitative; mixed methods	Narrative or pictorial assessment; views; attitudes; opinions	Qualitative, quantitative and mixed methods
Associated search terms	All African countries and regions listed separately; Africa; (keyword and MeSH term);	Initial search: Body image; thinness; body size	Not used in search	Body image; body perception; body esteem; body satisfaction; thinness OR associated MeSH terms	Not used in search
		Supplementary search. Body Image; body size; body weight; body esteem; weight control; body ideal; weight ideal; size ideal OR associated MeSH terms			
Boolean operators used	OR	OR	N/A	OR	N/A

Final Search Strategy: S AND PofI or E * NOT African-American AND *limited to humans.

### Search strategy

The SPIDER tool was used to define search terms and eligibility criteria^(34)^. The search was conducted on ASSIA, CINAHL, Medline, PsycINFO and Web of Science. Initial searches were conducted from 20/04/2015 until 15/05/2015. A supplementary search was then conducted, repeating the search strategies up to 31/08/2019. An example full search can be found in online Supplemental Material 1. Furthermore, the reference lists of studies included after full text screening were searched and topic experts consulted to identify additional studies.

### Screening

Duplicates were removed in MS Excel before screening. All papers were first screened on titles and abstracts and then on full text in MS Excel (OO, EC, RP, MH, CBN). Reasons for exclusion were recorded at full-text stage (i.e. no measure of body size preferences or factors influencing these; data not stratified by gender, ethnic group or country). To ensure consistency in the application of inclusion criteria, two authors (HO and CBN) checked 10 % of all excluded documents screened. There was a good degree of concordance overall; discrepancies were resolved through discussion.

### Data extraction

Data extraction of included studies was conducted in MS Excel by OO, EC, RP, MH, HO. Quantitative and qualitative data from mixed methods studies were extracted separately (see online Supplemental Material 2).

Data extracted from studies included general information (study ID, title, authors, date, study location (country, urban *v*. rural), study aim); study eligibility (participant selection, sample size, participant characteristics); method used to measure body image dimensions; ideal body size; body size self-assessment; body size self-satisfaction and factors influencing body size preferences. One author (RP) checked the full data extraction file to ensure accuracy and consistency across team members. Where data could not be extracted, authors were contacted to request relevant data, e.g. disaggregated data for pooled male and female samples.

### Quality appraisal

The ‘Standard Quality Assessment Criteria for Evaluating Primary Research Papers from a Variety of Fields’ (QualSyst) was used to critically appraise both the quality of the studies and their reporting^(35)^. This tool was chosen because it provided a standard checklist for all study designs^(35)^. Each study included in the review was rated using a predefined list of criteria (*n* 14 for quantitative studies, *n* 10 for qualitative studies). The original tool was modified by replacing the score for each criterion (0, 1, 2) with a qualitative assessment of high quality/green (low risk of bias), medium quality/yellow or low quality/red (high risk of bias) as the Cochrane guidance advises against the use of scores^(36)^. Five authors (OO, EC, RP, MH, HO) independently conducted quality appraisal and this was double-checked by RP and EC. Discrepancies in the rating were discussed until agreement was reached.

### Data synthesis

**Fig. 1:**
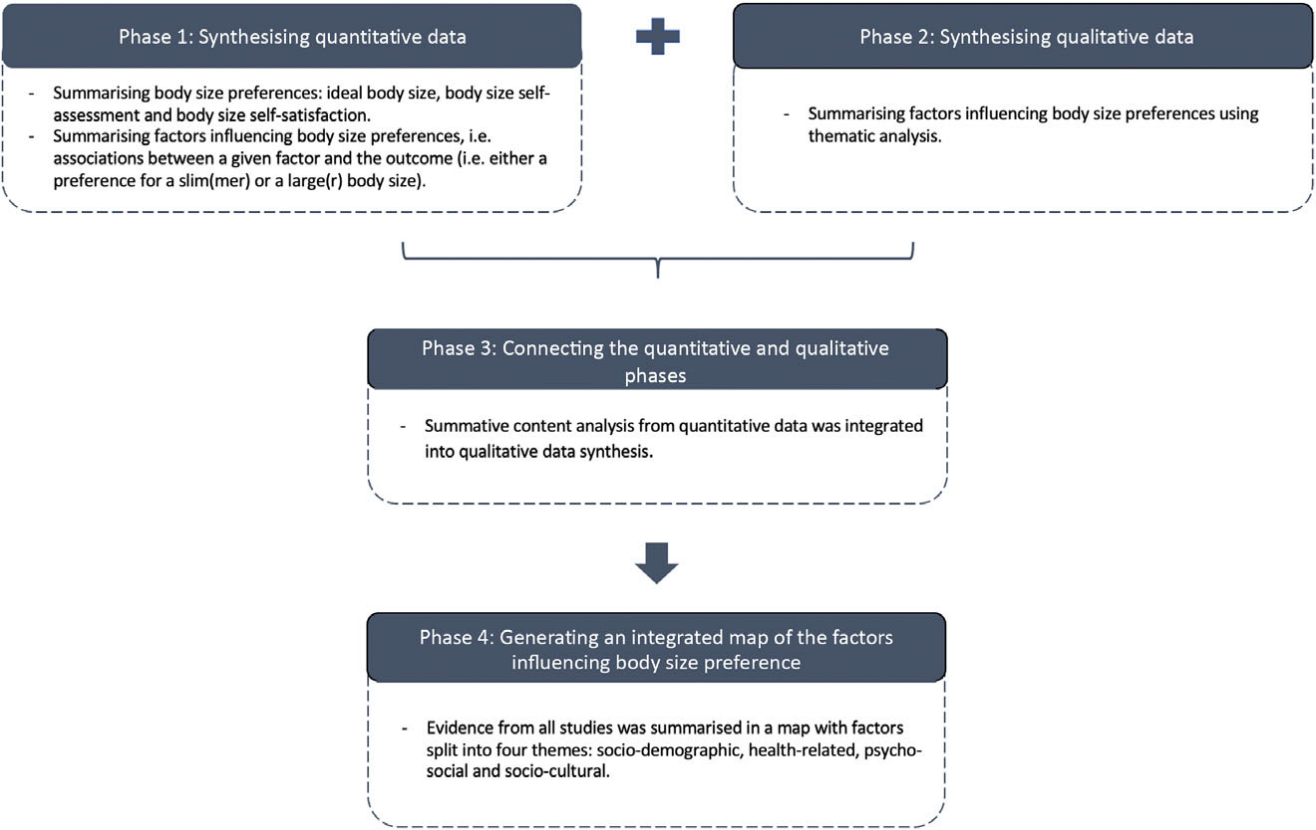
Visual model for mixed methods sequential explanatory design procedures

A sequential-explanatory approach was used to integrate quantitative and qualitative evidence^(37,38)^ (Fig. 1). In phase one, quantitative studies were synthesised to define African females’ body size preferences and the factors influencing these. Phase two involved summarising the evidence from qualitative studies on factors influencing body size preferences, and phase three aimed at integrating findings from both quantitative and qualitative studies. The qualitative data synthesis provided contextual and cultural understanding of the quantitative data. Phase four focused on generating an integrated (quantitative and qualitative evidence) visual map to represent factors associated with body size preferences.

#### Phase 1: Summarising body size preferences quantitatively and factors influencing these

To identify and summarise body size preferences, we focused on three specific body image dimensions: ideal body size, body size self-assessment and body size self-satisfaction^(39)^.

Ideal body size, assessed with various body image (i.e. figural/photographic rating) scales in several quantitative and mixed methods studies, was synthesised graphically where possible. To facilitate the comparison between studies using different scales, females’ ideal body size mean and males’ ideal body size mean for women and adolescent girls were converted into the four main BMI categories: underweight, normal weight, overweight and obesity, through the Silhouette Photographs scale presenting a convenient body image metric^(40)^.

For scales with real BMI values^(40–42)^, we matched BMI categories with each silhouette. For scales without real BMI values, we assigned a BMI category to each silhouette according to the BMI classification defined by authors of the selected articles below. For all articles using the Figure Rating Scale (FRS)^(43)^, the Body Image Instrument^(44)^, the Contour Drawing Rating Scale^(45)^, the Body Image Assessment for Obesity^(46)^, the Ideal Body Subscale^(47)^, the Figural Stimuli^(48)^, the Body Silhouette Chart^(49)^ and the Body Size Silhouettes^(50)^, we used respectively the BMI classification stated by Matoti-Mvalo and Puoane^(24)^, Pulvers *et al.* ^(44)^, Thompson and Gray^(45)^, Ettarh *et al.* ^(51)^, Cogan *et al.* ^(52)^, Duda *et al.* ^(48)^, Caradas *et al.* ^(53)^ and Rguibi and Belahsen^(8)^ (see online Supplemental Material 3). We extracted the BMI categories preferred by participants, defined from the ideal body size mean and its sd measured with the body image scales.

Studies that measured ideal body size using a questionnaire were summarised narratively. The second (i.e. body size self-assessment) and third (i.e. body size self-satisfaction) dimensions of body image were also summarised narratively.

To identify factors influencing body size preferences quantitatively, we summarised the associations between a given factor and the outcome (i.e. either a preference for a slim(mer) or a large(r) body size).

#### Phase 2: Summarising qualitatively factors influencing body size preferences

Thematic synthesis was used to summarise qualitative studies^(54,55)^. Themes, defined as concepts that occurred frequently across more than one article, were identified from the analysis of the extracted text.

#### Phase 3: Connecting the quantitative and qualitative phases

Summative content analysis^(56)^ from quantitative data was integrated into qualitative data synthesis. Factors identified from the quantitative and qualitative synthesis were grouped under four main overarching categories: socio-demographic, health-related, psycho-social and socio-cultural. These categories were defined based on the findings that emerged.

#### Phase 4: Generating an integrated map of the factors influencing body size preferences

Evidence from all studies was summarised in a map with factors split into the four categories described above.

## Results

### Description of studies

**Fig. 2:**
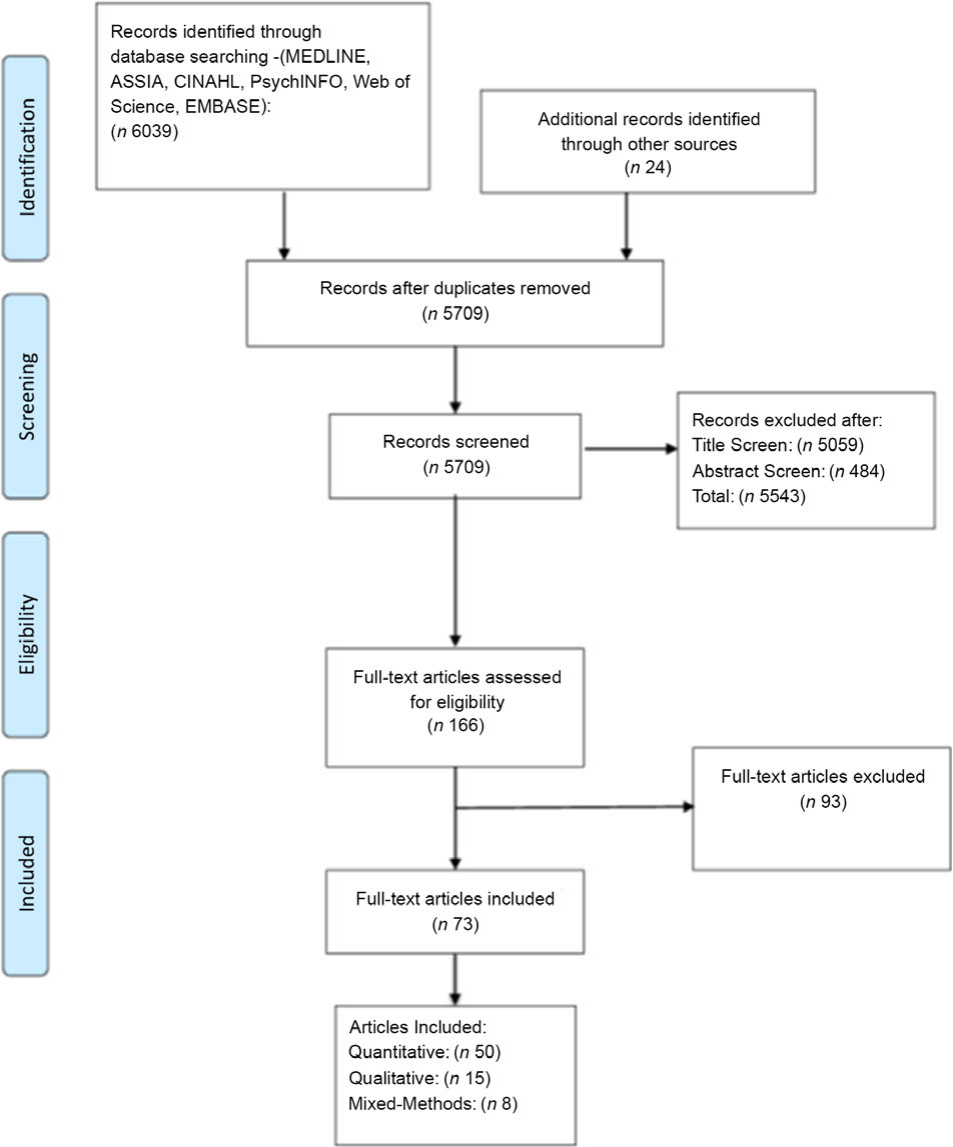
Preferred reporting items for systematic reviews and meta-analyses flow diagram showing the selection of studies for the present systematic mixed-methods review

**Fig. 3:**
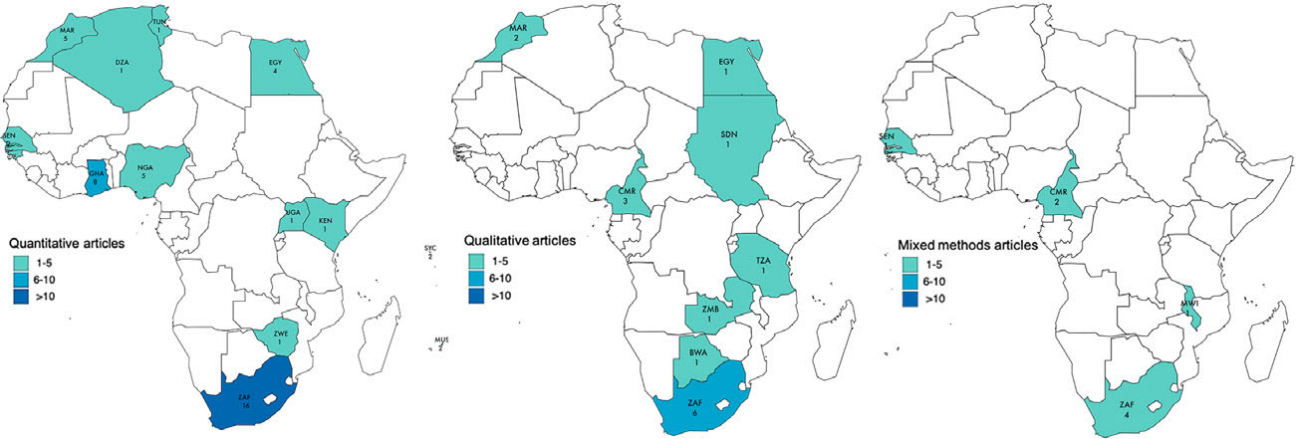
Map displaying the African countries included in the review

Seventy three articles were included (Fig. 2 ^(57)^). Studies were conducted in 21/54 African countries and clustered particularly within three regions: Southern Africa, West Africa and East Africa, with a predominance in South Africa (Fig. 3). More studies took place in urban (*n* 51) than rural (*n* 7) settings; and 15 studies included data from both settings.

Of the seventy-three articles, fifty were quantitative^(8,9,27,28,40,41,48,51–53,58–97)^, fifteen were qualitative^(11,23,98–110)^ and eight mixed methods^(24,42,111–116)^ (Table 2). All quantitative studies utilised a cross-sectional design, with the exception of one longitudinal design^(87)^. A total of 25 512 females and 2090 males aged ≥10 years from 17 African countries were included. Twenty-three qualitative studies from ten countries were included. Data from 828 participants and twenty households aged 10–70 years were synthesised (sample sizes between 10 and 193). Samples across studies were diverse in terms of age, SES, ethnicity, education and BMI.

**Table 2:** Description of included studies

Study ID	Country	Income level	Setting	Sample characteristics			Sample size
				Gender	Age group*	Mean age (sd)/age range (years)	
Quantitative articles (*n* 50)							
Salokun & Toriola^(58)^	Nigeria	Low	Urban	Females	Adolescents	12–15	140
Ford *et al.* ^(59)^	Egypt	Medium	Urban	Females	Adolescents/Young adults	19·5 (1·5)	160
Walker *et al.* ^(60)^	South Africa	Medium	Urban/Rural	Females	Adolescents	16·5 (1·9)	818
Furnham & Baguma^(61)^	Uganda	Low	Urban	Females	Adolescents/Young adults/Adults	22·3 (3·9)	51
Cogan *et al.* ^(52)^	Ghana	Medium	Urban	Mixed	Young adults	24·6 (no sd)	349 (♀: 184; ♂: 165)
Toriola *et al.* ^(62)^	Nigeria	Low	Urban	Females	Adolescents/Young adults/Adults	Students: 23·1 (3·3); Teachers: 35·0 (6·5)	151 (103 students; 48 teachers)
Caradas *et al.* ^(53)^	South Africa	Medium	Urban	Females	Adolescents	16·3 (1·1)	60
Jackson *et al.* ^(63)^	Egypt	Medium	Urban/Rural	Females	Adolescents	14·4 (1·7)	340
Holdsworth *et al.* ^(40)^	Senegal	Low	Urban	Females	Young adults/Adults/Middle-aged	20–50	301
Singh^(64)^	Guinea Bissau	Low	Rural	Females	Adults/Middle-aged	20–55	37
Faber & Kruger^(65)^	South Africa	Medium	Rural	Females	Young adults/Adults	33·5 (8·9)	187
Mciza *et al.* ^(66)^	South Africa	Medium	Urban	Females	Young adults/Adults/Middle-aged	40 (10·7)	63
Duda *et al.* ^(67)^	Ghana	Medium	Urban	Females	All	35·9 (19–74)	305
Rguibi & Belahsen^(8)^	Morocco	Medium	Urban	Females	All	>15	249
Rguibi & Belahsen^(9)^	Morocco	Medium	Urban	Females	Adults/Middle-aged	36·8 (11·8)	249
Siervo *et al.* ^(68)^	Gambia	Low	Urban	Females	Adolescents/Young adults/Adults	Adolescents: 18·6 (3·4); Adults: 42·5 (5·2)	100
Szabo & Allwood^(69)^	South Africa	Medium	Urban/Rural	Females	Adolescents	15·9 (2·1)	939
Duda *et al.* ^(48)^	Ghana	Medium	Urban	Females	All	35·9 (19–74)	305
Jumah & Duda^(70)^	Ghana	Medium	Urban	Mixed	Young adults/Adults	33·0 (11·5)	185 (♀: 62; ♂: 123)
Frederick *et al.* ^(71)^	Ghana	Medium	Rural	Mixed	Adolescents/Young adults/Adults	27·8 (10·3)	48 (♀: 26; ♂: 22)
Lahmam *et al.* ^(72)^	Morocco	Medium	Rural	Females	Adults/Middle-aged	50·0 (16·3)	271
Okoro & Oyejola^(73)^	Nigeria	Low	Urban	Mixed	Adults/Middle-aged	55·7 (12·9)	139 (♀: 84; ♂: 55)
Mwaba & Roman^(74)^	South Africa	Medium	Urban	Females	Adolescents	19·2 (no sd)	150
Swami *et al.* ^(27)^	South Africa	Medium	Urban/Rural	Mixed	Adults/Middle-aged	39·3 (10·7)	205 (♀: 108; ♂: 97)
Alwan *et al.* ^(75)^	Seychelles	High	Urban/Rural	Females	Adolescents	14·1 (1·5)	476
Coetzee & Perrett^(76)^	South Africa	Medium	Urban	Females	Adolescents/Young adults/Adults	18–30	53
Mchiza *et al.* ^(77)^	South Africa	Medium	Urban	Females	Adolescents/Adults/Middle-aged	Daughters: 10·5 (0·9); Mothers: 38·5 (9·0)	44
Benkeser *et al.* ^(78)^	Ghana	Medium	Urban	Females	Adults/Middle-aged	46·3 (18·2)	2814
Maruf *et al.* ^(79)^	Nigeria	Low	Urban	Females	Young adults	21·8 (1·8)	64
Swami *et al*.^(41)^	Zimbabwe	Low	Urban	Females	Adolescents/Young adults/Adults	25·3 (6·9)	140
Bhurtun & Jeewon^(80)^	Mauritius	High	Urban/Rural	Females	Adolescents	13–18	90
Ettarh *et al.* ^(51)^	Kenya	Medium	Urban slums	Females	Adults	42·0 (no sd)	2265
Jafri *et al*.^(81)^	Morocco	Medium	Urban	Females	All	≥ 18	425
El Ansari *et al.* ^(82)^	Egypt	Medium	Urban	Females	Adolescents	18·6 (1·2)	1663
Gitau *et al.* ^(85)^	South Africa	Medium	Urban	Mixed	Adolescents	13 and 17	1302 (♀: 675; ♂: 627)
Gitau *et al.* ^(83)^	South Africa	Medium	Urban	Females	Adolescents	15·0 (no sd)	183
Gitau *et al*.^(84)^	South Africa	Medium	Urban	Males	Adolescents	14·9 (no sd)	179
Musaiger & Mannai^(28)^	Egypt	Medium	Urban	Females	Adolescents/Young adults/Adults	17–32	210
Okoro *et al.* ^(73)^	Nigeria	Low	Urban	Mixed	Adults/Middle-aged	43·9 (17·2)	524 (♀: 304; ♂: 220)
Gradings *et al.* ^(87)^	South Africa	Medium	Urban	Females	Adults/Middle-aged	Baseline: 41·1 (5·4); Follow-up: 49·3 (5·3)	430
Amenyah & Michels^(88)^	Ghana	Medium	Urban	Mixed	Adolescents	11–18	370 (♀: 194; ♂: 176)
Caleyachetty *et al.* ^(89)^	Mauritius	High	Urban/Rural	Females	Adults/Middle-aged	45·8 (35·0–56·7)	3022
Gualdi-Russo *et al.* ^(90)^	Tunisia/Morocco	High/Medium	Urban	Females	All	Tunisians: 28·7 (11·5); Moroccans: 39·5 (13·1)	228 (104 Tunisian; 124 Moroccan)
Pedro *et al.* ^(91)^	South Africa	Medium	Rural	Females	Adolescents	13·6 (1·2)	195
Yepes *et al.* ^(92)^	Seychelles	High	Urban/Rural	Females	Young adults/Adults/Middle-aged	24–64	709
Macia *et al.* ^(93)^	Senegal	Low	Urban/Rural	Mixed	Young adults/Adults/Middle-aged	36·2 (13·9)	1500
Michels & Amenyah^(94)^	Ghana	Medium	Urban	Females	Adolescents	11–18	194
Prioreschi *et al.* ^(95)^	South Africa	Medium	Urban/Rural	Females	Adolescents/Young adults	20·6 (1·1)	1019
Naigaga *et al*.^(96)^	Algeria	High	Urban	Females	All	18–45 and ≥ 46	180
Cohen *et al*.^(97)^	South Africa	Medium	Urban	Females	Adolescents/Adults/Middle-aged	Daughters:17·9 (0·4); Mothers: 41·4 (8·0)	1230 (615 mothers; 615 daughters)
Qualitative articles (*n* 15)							
Mvo *et al.* ^(98)^	South Africa	Medium	Urban slums	Female	Adolescents/Young adults/Adults	18–36	10
Treloar *et al.* ^(99)^	Cameroon/Egypt	Medium	Urban/Rural	Mixed	Not reported	Not reported	11 (♀: 6; ♂: 5)
Kiawi *et al.* ^(100)^	Cameroon	Medium	Urban	Female	All	≥15	27
Dapi *et al.* ^(101)^	Cameroon	Medium	Urban/Rural	Female	Adolescents	12–15	15
Batnitzky^(102)^	Morocco	Medium	Urban	Female	Not reported	Not reported	20 households
Ezekiel *et al*.^(23)^	Tanzania	Low	Rural	Mixed	Adolescents/Young adults	17·2 (no sd)	193 (♀: 84; ♂: 109)
Batnitzky^(11)^	Morocco	Medium	Urban	Female	Not reported	Not reported	20
Shaibu *et al.* ^(103)^	Botswana	High	Urban	Mixed	Not reported	Not reported	18 stakeholders
Morris & Szabo^(104)^	South Africa	Medium	Urban	Female	Adolescents	14–18	40
Ahmed & Saltus^(105)^	Sudan	Low	Urban	Female	Adolescents/Young adults	16–25	19
Draper *et al.* ^(106)^	South Africa	Medium	Urban slums	Female	Young adults/Adults/Middle-aged	24–51	21
Pradeilles^(107)^	South Africa	Medium	Urban slums	Mixed	Young adults/Adults	20–39	51 (♀: 19; ♂: 32)
Okop *et al.* ^(108)^	South Africa	Medium	Urban slums	Female	Adults/Middle-aged	35–70 years	36
Phillips *et al*.^(109)^	South Africa	Medium	Urban slums	Female	Young adults/Middle-aged	Daughters: 24·2; Mothers: 53·0	32 (17 daughters; 15 mothers)
Tateyama *et al.* ^(110)^	Zambia	Medium	Rural	Female	Adults/Middle-aged	≥40	40
Mixed methods articles (*n* 8)							
Puoane *et al*.^(111)^	South Africa	Medium	Urban slums	Female	Adults/Middle-aged	43·2 (7·2)	Quant: 44; Qual: 27
Bodiba *et al.* ^(112)^	South Africa	Medium	Urban	Female	Adolescents	16 (17–19)	Quant: 75; Qual: not reported
Puoane *et al*.^(113)^	South Africa	Medium	Urban slums	Female	Adolescents	10–18	Quant: 240; Qual: 60
Matoti-Mvalo & Puoane^(24)^	South Africa	Medium	Urban slums	Female	All	18–65	Quant: 513; Qual: 20
Cohen *et al.* ^(42)^	Cameroon	Medium	Urban	Mixed	All	Quant: 35·2 (13·5; Qual: 18–30 or 50–65	Quant: 181 (♀: 98; ♂: 83); Qual: 16 (balanced sex ratio)
Cohen *et al.* ^(114)^	Cameroon	Medium	Urban/Rural	Mixed	All	Quant: 38·6 (13·6); Qual: 18–30 and 50–65	Quant: 577 (♀: 339; ♂: 238); Qual: 24 (♀: 12; ♂: 12)
Croffut *et al*.^(115)^	Malawi	Low	Urban/Rural	Female	Young adults/Adults	27·0 (5·3)	Quant: 64; Qual: 64
Cohen *et al.* ^(116)^	Senegal	Low	Urban/Rural	Mixed	Adolescents/Young adults/Middle-aged	Quant: 36·3 (15·6); Qual: <25 and >45	Quant: 597 (♀: 313; ♂: 284); Qual: 84 (♀: 42; ♂: 42)

♀: females; ♂: males.

* Age group: adolescents: 10–19; young adults: 20–25; adults: 25–44 and middle-aged: 45+.

### Quality appraisal

The quality assessment (see online Supplemental Material 4) revealed that quantitative and qualitative studies separately scored highly on criteria such as question/objective sufficiently described, evident and appropriate study design and conclusions. Quantitative studies scored highly on well-defined outcome measures; however, some studies reported poorly on subject selection and characteristics, estimate of variance and control for confounding. Similarly, in almost all qualitative studies, authors failed to show reflexivity and/or to report verification of the procedure to establish credibility.

### Data synthesis

#### Body size preferences for African women and adolescent girls

##### Dimension one: ideal body size

The majority of quantitative and mixed methods studies (46/58 (79·3 %)) assessed African women’s body size ideals (see online Supplemental Material 5). Most studies used body image scales whilst the remaining studies used questionnaires to capture the relationship between specific attributes (e.g. healthy, wealthy, dignified, respected) and different body sizes.

**Fig. 4:**
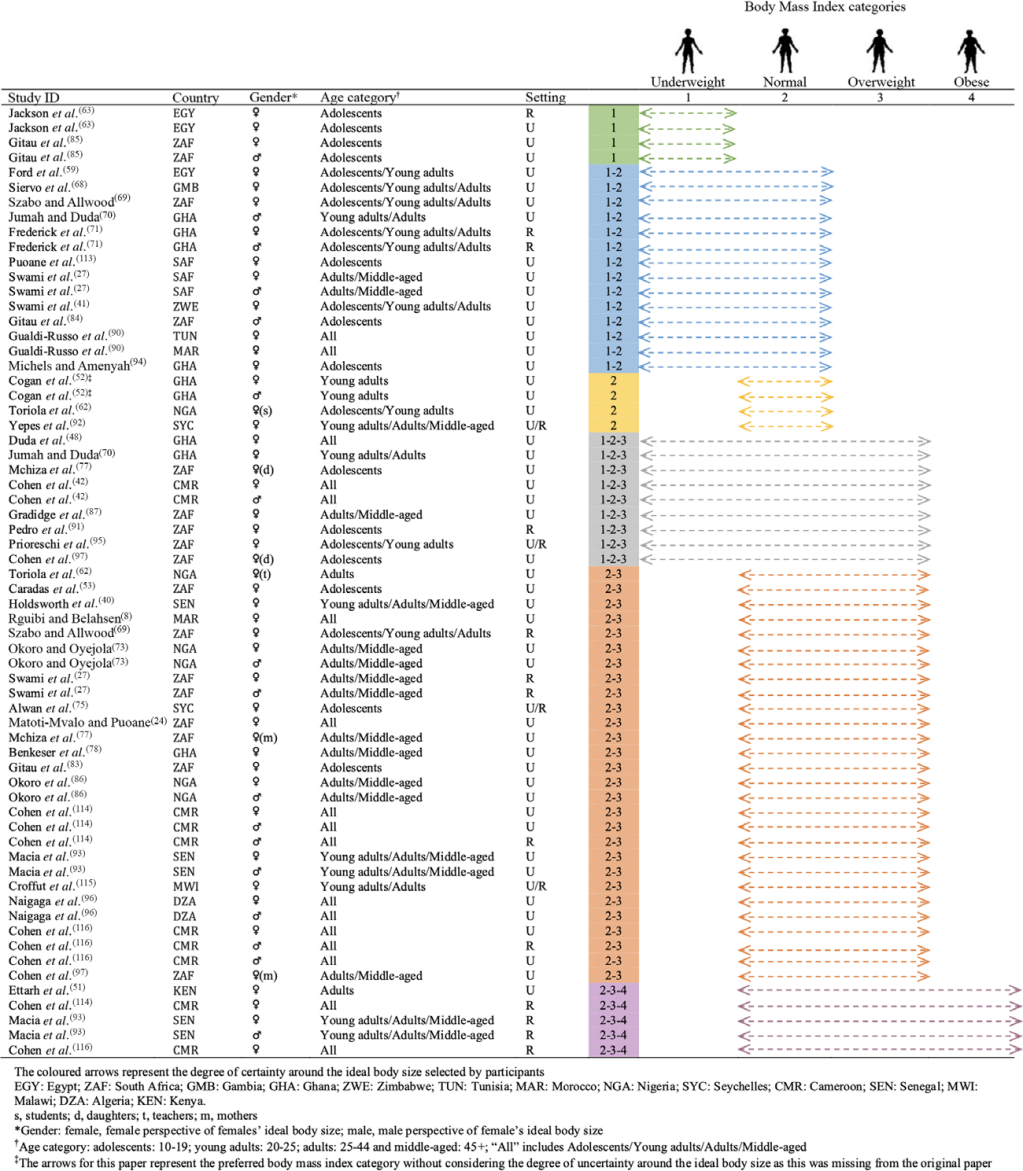
Body size ideals for African women and adolescent girls

For studies using body image scales and for which a BMI category could be extracted, we found that, overall, participants in most studies preferred normal weight to overweight (Fig. 4). We observed a positive relationship between age category and ideal body size, with valorisation of underweight in some adolescent females^(63,85)^ and valorisation of overweight in middle-aged women^(27,73,77,78,86,97)^. The rural–urban comparison showed that the ideal BMI category was higher in rural than urban areas in South Africa^(27,69)^, Senegal^(93,116)^ and Cameroon^(114)^.

Few studies explored attributes associated with different body sizes. One study^(66)^ observed differences between urban black and white South African women, especially in middle-aged women, regarding the ‘normal’ and ‘fat’ attributes; and observed that black girls and their mothers were less likely to associate ‘fatness’ with being unhappy in comparison with the white and mixed ancestry group. Likewise, another study^(65)^ found that more than 80 % of black South African rural women disagreed that ‘fat people eat more than thin people’. Moreover, 25 % of these women associated overweight with a lack of financial problems, and more women with a normal weight associated overweight/obesity with food intake, compared with those overweight and obese. In a black township of Cape Town, 74 % of black females considered that being fat ‘made you dignified’ and 43 % believed that this weight status led one to ‘feel better about yourself’^(111)^. Indeed, a woman who was overweight was perceived as ‘well-liked’ (100 %), ‘proud of her movements’ (100 %), ‘healthy’ (100 %) and ‘happy’ (94 %). However, a woman who was ‘thin’ was perceived by 33 %, 58 % and 60 % of the sample as ‘sick’, a ‘woman with worries’ and ‘not treated well by her husband’, respectively.

##### Dimension two: body size self-assessment

In total, 19/58 (32·7 %) studies included information on body size self-assessment (see online Supplemental Material 5). Using body image scales, one study^(51)^ found that 34·6 % of Kenyan women living in urban Nairobi underestimated their body weight; with 28·8 % of women who underestimated their weight classified as obese, a pattern also observed in Cameroonian urban women^(42)^. The same was also observed with overweight/obesity in Nigerian students and South African black urban women, as well as in black schoolgirls, Algerian Saharawi refugees, urban Tunisian, Moroccan and Malawian women^(24,53,79,87,90,96,97,115)^. Only 37·1 % of Ghanaian women living in Accra perceived their current body size as overweight or obese, even though 49·9 % were in these weight categories^(70)^, a pattern also observed by others in the same city^(48,78)^.

Using questionnaire items in rural Morocco, almost all (99·2 %) women who were overweight/obese underestimated their body weight status, which increased with age^(72)^. Similarly, 89 % of middle-aged South African black women living in Cape Town were happy with their weight, whereas most of them were overweight or obese^(66)^. Likewise, approximately two-thirds of Black women with overweight/obesity in Cape Town did not perceive themselves as such^(111)^. Finally, in urban Cameroon, 37·5 % of women considering themselves normal weight were overweight or obese^(42)^.

Using body image scales, most adolescent Egyptian schoolgirls estimated their body weight accurately along the spectrum of BMI categories^(63)^. Similarly, in the Seychelles, 24 % of adolescent girls of normal weight considered themselves overweight,^(75)^ and in Mauritius most adolescent girls who classified themselves as overweight were in fact normal weight^(80)^.

##### Dimension three: body size self-satisfaction

Overall, 44/58 (75·9 %) studies included information on body size self-satisfaction (see online Supplemental Material 5).

Of the studies that assessed satisfaction using scales (*n* 24), seventeen found a positive Feel minus Ideal Discrepancy (FID) (i.e. current > ideal), meaning that women and/or adolescent girls wanted to lose weight^(27,42,48,53,59,66,69,71,73,79,87,90,91,95,97,114,116)^. A further five studies found a negative FID (i.e. current < ideal), meaning that women and or/adolescent girls wanted to gain weight^(41,52,62,86,115)^ and two found mixed results. The two studies reporting mixed results included Mchiza *et al.* ^(77)^ with a positive FID among mothers and null FID (i.e. satisfied with current weight) among black girls, and Siervo *et al.* ^(68)^ with a positive FID among middle-aged women and a negative FID among young women.

Of the studies that assessed satisfaction using questionnaires (*n* 30), ten found that women who were overweight or obese were satisfied with their current body size^(8,40,51,60,65,75,78,80,88,97)^. The percentage of participants with a BMI ≥ 25 kg/m^2^ who were satisfied with their current weight ranged from 10·5 % (obese women only)^(40)^ to 95·0 % (overweight women only)^(65)^. For all papers reporting prevalence of body satisfaction among women who were overweight or obese separately, we found that the percentage of satisfaction was higher among women who were overweight, indicating that they were overall more satisfied with their body size in comparison with women who were obese^(8,40,51,65,78)^. One study conducted in South Africa^(97)^ found that the level of body satisfaction was slightly higher among mothers (28·0 %) when compared with their daughters (23·1 %) (both had a BMI ≥ 25 kg/m^2^), and another study in South Africa^(60)^ showed that body satisfaction was higher among women who were overweight or obese in rural areas (61·4 %) compared with those in urban areas (32·3 %). Eleven studies reported information on the proportion of participants with a BMI ≥ 25 kg/m^2^ wanting to be larger^(8,40,51,65,72,75,78,80,88,96,97)^. We found that this phenomenon ranged from none in the Seychelles^(75)^, Mauritius^(80)^ and Algeria^(96)^ to 45·5 % in Morocco^(72)^.

Of the studies that assessed satisfaction using questionnaires (*n* 30), eight found underweight participants (BMI < 18·5 kg/m^2^) were satisfied with their current body weight^(8,40,51,60,78,80,88,97)^. The proportion of participants satisfied ranged from 2·3 % amongst women in urban Ghana^(78)^ to 95·5 % amongst adolescent girls in rural South Africa^(60)^. A study comparing body satisfaction among mothers and daughters who had a BMI < 18·5 kg/m^2^ showed that daughters were more satisfied with their body size (45·9 %) in comparison with mothers (22·2 %), meaning that younger generations might have a preference for slimmer bodies^(97)^. Six studies reported information on the proportion of participants with a BMI < 18·5 kg/m^2^ willing to lose weight^(8,9,40,78,80,97)^. We found that the prevalence of women who were underweight and wanted to be slimmer ranged from none in Morocco^(8,9)^ to 33·3 % in Mauritius^(80)^.

### Factors influencing body size preferences: quantitative and qualitative evidence

**Fig. 5:**
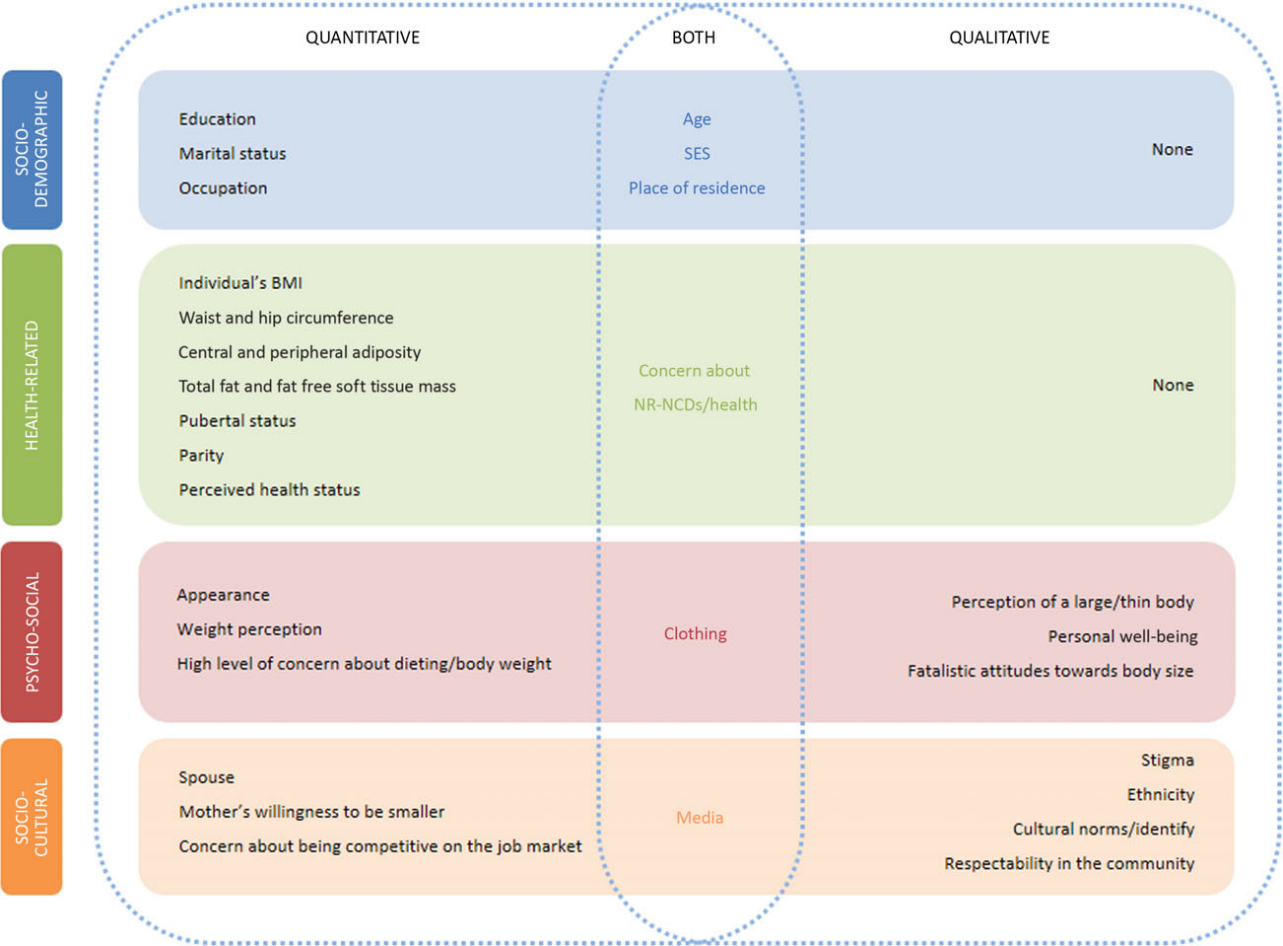
Integrated map of the factors influencing body size preferences for African women and adolescent girls

Twenty-nine quantitative studies and twenty-three qualitative studies (including eight mixed-methods studies) reported factors associated with body size preferences (see online Supplemental Material 5). For both types of studies, factors were grouped into four overarching themes (socio-demographic, health-related, psycho-social and socio-cultural), and their influence on preferred body size (i.e. preference for a slim(mer) or a large(r) body size) indicated (Tables 3 and 4). Eleven analytical themes emerged from qualitative studies (Table 5). The integrated results are discussed below and summarised in Fig. 5.

**Table 3:**
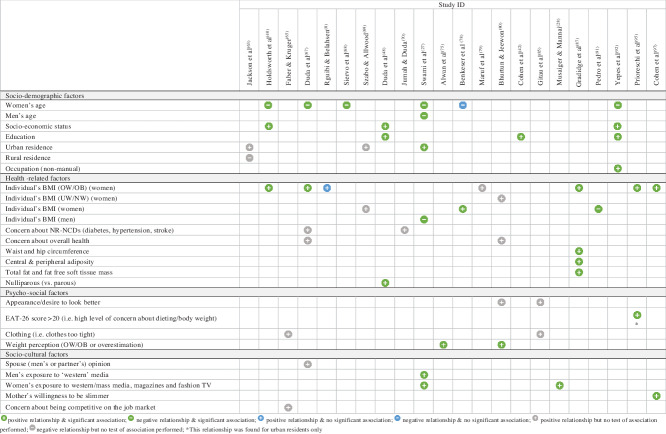
Factors associated with a preference for a slim(mer) body size among African women and adolescent girls from the quantitative evidence synthesis

**Table 4:**
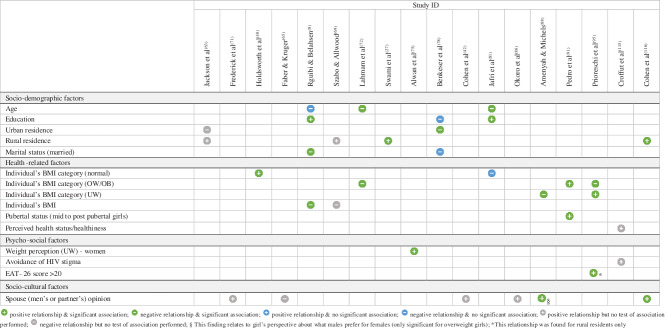
Factors associated with a preference for a large(r) body size among African women and adolescent girls from the quantitative evidence synthesis

**Table 5:** Factors influencing body size preferences for African women and adolescent girls from the qualitative evidence synthesis

Theme	Child theme	Grand-child theme	Studies
Sociodemographic factors			
Place of residence	Integration into urban lifestyle	Urban lifestyle and weight gain/valorisation of overweight/stoutness	116
	Rural residency	Rural and limited corpulence valued (high fatness not adapted to rural labour)	116
		Rural and valorisation of stoutness	114
Socio-economic status	SES	Higher SES have thin body ideal	11,102
Age	Young	Young people have thin(ner) body ideal	107,109,116
	Old	Middle-aged and older women tend to value overweight/heavier body sizes	
Health-related factors Health	Fatness and health	Fatness and NR-NCDs (e.g. hypertension, diabetes)/poor physical health	24,100,103,106,107,108 *,^109^,^110^,^111^,^113^,^115^
Psychosocial factors			
Appearance and attractiveness (to men and/or society)	Perception of a large/thin body	Stoutness and beauty (pre-marriage)	114
		Fatness and having a figure/good shape	23,24,42,101,106,107,113,105
		Full-figured (medium-sized) women who were neither slim nor fat were seen as attractive by society.	105
		A full-figured body was considered to be sexually attractive, enhancing sexual gravitation	105
		Larger body sizes/fatness, chubbiness and big body size as signs of health	107,108 †,^115^
		Obesity unattractive	100,105,112
		Thinness and beauty	100,104,106,107,108,110
		Thinness and good shape	107,108 *
		Thinness and intelligence	108
		Thinness – fashionable clothes	104,106,112,113
		Thinness and vulnerability/proneness to diseases	105,109
		Thinness and healthiness	109
		Thinness unattractive	107
	Clothing	Clothing fitting properly – looking nicer on full-figured women	105,114,115
		Thinness – fashionable clothes	114,106,112,113
		Clothing fitting properly – looking nicer on a thinner body size	98,100,104,108,111
		Thinness and looking smart	100,108
Personal well-being	Personal well-being	Stoutness and well-being	114
		Full-figured and social esteem	105
		Fatness and happiness	107,108,109
		Obesity limitations – difficulty performing exercise and daily activities, shortness of breath	11,98,100,102,108,110,112,113
		Thinness – feeling better about oneself	106,111,112
		Thinness and happiness	108 *
		Thinness and physically active	107
		Thinness and popularity	111,112
		Thinness and unhappiness	108 ‡
		Thinness and stress/depression	108
		Thinness and laziness	110
Fatalistic attitudes toward body size	Aetiology of thinness/fatness	Body size is God given	11,100,102
		Body size is driven by genetic	100,108,113
		Fatness caused by pregnancy	11,102,110
		Fatness caused by medication (e.g. ART treatment)	98,110
		Overweight caused by inability to afford good quality meat	111
		Obesity attributed to laziness, sluggishness, stigma and tiredness	108
		Fatness and unhealthy eating (both in quality and quantity)	98,99,106,108, 111,113
		Weight gain from stress	98,99,111
		Thinness and eating too little or not healthily	107,108
		Weight loss from stressful times (e.g. stress, financial problems, social problems, physical abuse, drug abuse)	98,104,108
Socio-cultural factors			
Negative treatment by the community	Thinness and misfortune	Thinness and poverty/failure in life	42,100,106
		Thinness and perception that family cannot afford food	105
	Stigma	Thinness/weight loss associated with sickness, e.g. HIV/AIDS, TB, cancer, anorexia	23,24,100,103,104,106,107,108,110,111,113
		Social discrimination against overweight	98,103,104,112,113
Acculturative stress	Media	Thinness and modelling	101,104,113
		Media promotion of thin and thinness	104,112,116
	Ethnicity	Pressure from other ethnic groups to be thin	98,104
Happy to accept African heritage	Cultural norms/identify	Fatness as norm/African heritage - Full figured preservation of identity	42,104,105,106,107,108,110,112
		Thinness classed as ‘un-African’	107
Social Standing	Respectability in community	Fatness and doing well in life	23,24,42,99,100,103,111,113,114
		Fatness and increased chances of getting married	109
		Body size after marriage/fatness or weight gain after marriage symbol of the capacity of the husband to look after his family	99,100,111,114
		Stoutness/fatness and fertility	107,114
		Fatness and ability to take good care of family	24,99,110,114
		Fatness and dignity/respect for a woman and her family	107,108
		Fatness as a symbol of wealth and prosperity/full figured and economic standing	105,108,110,114,116
		Fatness and capability/strength	23,24,42,101, 107,111,113
		Thinness and strength	107
		Thinness and affluence	11,102

* Found for normal weight women only.

† Found in overweight/obese women.

‡ Found in obese women.

### Socio-demographic factors

The quantitative evidence showed that increased SES^(40,48,92)^, non-manual occupations^(92)^ and living in urban areas^(27,63,69)^ were associated with slimmer body size preferences. Rural residency was associated with a greater preference for a larger body size,^(27,63,69,116)^ whilst marriage was associated with a lower preference for a larger body size^(8,78)^. Younger participants had a greater preference for slimmer body sizes in Senegal^(40)^, Ghana^(67,78)^, Gambia^(68)^, South Africa^(27)^ and the Seychelles^(92)^, but a greater preference for a larger body size in three studies conducted in Morocco^(8,72,81)^. Increased years of formal education was associated with a greater preference for both slimmer body size ideals in Ghana^(48)^, Cameroon^(41)^ and Seychelles^(92)^ and larger body size ideals in Morocco^(8,81)^. However, one study^(78)^ in Ghana found that increased years of formal education was associated with a lower preference for larger body size but this was NS.

The qualitative evidence revealed that higher SES^(11,102)^ and younger participants^(107,109,116)^ had slimmer body size preferences. Valorisation of stoutness was found in both urban Senegal^(116)^ and rural Cameroon^(114)^, especially in middle-aged and older women. However, high fatness was not valued by younger and also older women in rural Senegal, as it was deemed ill-adapted to rural labour and hence, not valued^(116)^.

‘*If you are too rey [stout] in the village, you will not be able to work or cultivate*…’ (middle-aged woman, urban, Senegal)^(116)^

### Health-related factors

#### BMI

Individual BMI was associated with body size preference, although mixed results were found.

Overall, increased BMI in women was positively associated with a greater preference for slimmer body sizes^(69,78)^, with the exception of one study^(91)^, and negatively associated with a preference for a large(r) body size^(8,69)^. One study also found a negative association between men’s individual BMI and a preference for slimmer female body sizes^(27)^.

Women who were overweight or obese had a predominantly greater preference for a slimmer body size^(8,40,67,79,87,95,97)^. Similarly, a greater waist and hip circumference, central and peripheral adiposity and total fat and fat free soft tissue mass were positively associated with slimmer body size preferences or participants desire for weight loss^(87)^. Being overweight or obese was also significantly associated with a lower preference for a larger body size^(72,95)^, with the exception of one study in South Africa, for which the association was positive^(91)^.

Women who were underweight or normal weight had either a greater preference for a slimmer body size/lower preference for a larger body size^(80,81,88)^ or a greater preference for a large(r) body size^(40,95)^.

#### Concern about health

Two quantitative studies found that participants who reported being concerned about their overall health status or the possibility of developing nutrition-related noncommunicable diseases had a greater preference for a slimmer body size^(67,70)^. The relationship between overweight/fatness and nutrition-related noncommunicable diseases was reported in eleven qualitative studies^(24,100,103,106–111,113,115)^. As a result, some participants wanted to lose weight for health reasons.

‘*I heard that when a person is fat there are a lot of fats and oils in the body, which may result in some diseases, like hypertension and diabetes*’…’*I would like to have medium [overweight] body, because sometimes when you are too fat, it becomes difficult to walk*’ (adult women, urban/rural, Malawi)^(115)^

#### Biological factors

Being nulliparous^(48)^ was associated with a greater preference for a slimmer body size whilst pubertal status (i.e. mid to post-pubertal girls)^(91)^ was associated with a preference for a larger body size.

### Psycho-social factors

#### Appearance and attractiveness

Quantitative studies reported a positive association between appearance or a desire to look better and a preference for a slimmer body size^(80,82)^. Likewise, clothing (i.e. clothes too tight) was positively associated with a desire for a slimmer body size^(69,83)^.

Appearance and attractiveness to men and/or society was a theme that emerged in most of the qualitative studies. An important aspect of appearance was the way clothing fell on different body sizes. Participants were motivated to lose weight to fit into their old clothes, to look smart or because they felt that clothes on thinner body sizes looked nicer^(98,100,104,108,111)^.

‘*Being overweight does not look nice, people often think that you are pregnant*’… ‘*Bigger clothes are more expensive and less pretty*’ (adult/middle-aged women, urban, South Africa)^(111)^

Four studies reported that fashionable clothes only came in smaller sizes and would therefore only fit slim people^(104,106,112,113)^. However, the importance of filling out clothes to showcase one’s figure was also reported in three studies^(105,114,115)^.

Body size was associated with physical attractiveness, which in turn equated to a better chance of ‘getting a boyfriend or husband’. In six studies, thinness was associated with beauty and good shape^(100,104,106–108,111)^, and three studies reported ‘fatness’ as unattractive and diminishing one’s chances of securing a boyfriend/husband^(100,105,112)^. On the other hand, one study associated stoutness with beauty in women pre-marriage^(114)^ and seven studies associated ‘being fat’, particularly having large hips, with ‘having a figure’ or a ‘good shape’^(23,24,41,101,106,107,113)^.

‘*Our culture says that we are supposed to be fat. You must have structure, you must be beautiful. In other words, our culture does not allow women to be thin. They say that someone who is fat is sexier’*. (adult/middle-aged woman, urban, South Africa)^(106)^

To have a good figure, it was seen as important that women gained weight proportionally and did not get too fat^(42)^. It was seen as particularly important that women were not physically restricted by their body size, otherwise their fatness shifted into the realm of being perceived as a result of greed or laziness. In another study, full-figured women (defined as women of a ‘medium size’, neither slim nor fat by investigators) were seen as attractive by society and were considered to be sexually attractive, enhancing sexual gravitation^(105)^. Body size was also associated with intelligence (thin women were perceived as smart)^(108)^ and health, but the evidence was mixed for the latter. In two studies, we found that thinness was associated with good health^(105,109)^ whilst in three other studies, we found that being overweight was a sign of perceived good health^(107,108,115)^.

#### Body size perception

An overestimation of body size or perception of self as overweight/obese was significantly associated with a preference for a slimmer body size^(75,80)^, whereas women who perceived themselves as underweight had a greater preference for a larger body size^(75)^. In one study^(95)^, an EAT-26 score >20, which indicates a high level of concern about dieting, body weight or problematic eating behaviours, was associated with either a greater preference for a slimmer body size (urban residents) or larger body size (rural residents).

#### Avoidance of HIV stigma

One quantitative study found a positive association between avoiding HIV stigma and a preference for a large(r) body size^(115)^.

#### Personal well-being

Perceptions attached to body size and personal well-being were discussed by participants in most of the qualitative studies. In five studies, fatness or a full-figured body size was associated with well-being^(114)^, social esteem^(105)^ and happiness^(107–111)^.

*‘If a person is fat [overweight], we usually assume she is happy and has a lot of money. It’s evident that he/she eats nicely’* (middle-aged woman, urban, South Africa)^(108)^

However, the difficulties associated with obesity (i.e. difficulty performing exercise and daily activities and shortness of breath) were also highlighted^(11,98,100,102,108,111–113)^.

*‘I think there is nothing good about being fat because you are constantly sick, and you constantly have pains’* (adolescent girl, urban, South Africa)^(113)^

The results on thinness were mixed with studies highlighting an association between thinness and markers of personal well-being, such as self-esteem^(106,111,112)^, happiness^(108)^, being physically active^(107)^ and popularity^(111,112)^ and other studies providing evidence of an association between thinness and unhappiness^(108)^, stress/depression^(108)^ and laziness^(110)^.

#### Fatalistic attitudes towards body size

Various reasons were proposed as ‘causing’ obesity, many of which are founded in scientific evidence, ranging from unhealthy eating in terms of quantity and quality^(98,99,106,108,111,113)^, stress^(98,99,111)^, medication (e.g. anti-retroviral treatment)^(98,110)^, genetics^(100,108,113)^, pregnancy^(11,102,111)^ and laziness^(108)^.

*‘There are people who are born fat even though they try to lose weight’* (adolescent girl, urban, South Africa)^(113)^

However, the belief that obesity was ‘God-given’ also emerged^(11,100,102)^:

*‘I used to be thin before I had children. Then slowly after each child I became bigger. This is what Allah intends for a mother to look like’* (woman, urban, Morocco)^(11)^

This fatalistic belief can lead to participants accepting their body size and being less motivated to lose weight^(11,100)^. Factors influencing thinness included stress^(98,104,108)^ and eating too little^(107,108)^.

### Socio-cultural factors

#### Family influence

From the quantitative evidence, males’ or husbands’ opinions^(42,65,67,71,86,88,116)^ and mothers’ opinions^(97)^ influenced women’s body size preferences. This was a positive influence for a preference for a slimmer body and mixed for a preference for a large(r) body size.

#### Negative treatment by the community

Thinness and/or weight loss were associated with misfortune in four studies. A person who was thin was seen as being too poor to afford food, or having failed in life^(42,100,105,106)^.

*‘My parents also put pressure on me, always commenting on my weight and why I look so thin. They think that being thin is a stigma as people might think that the family cannot afford to buy food’* (adolescent girl, urban, Sudan)^(105)^

Activities associated with trying to lose weight, such as walking, were also associated with poverty^(100,106)^.

Being thin or losing weight was also strongly associated with illness, particularly with HIV and/or TB^(23,24,100,103,104,106–108,110,111,113)^. Participants preferred not to embody these factors – poverty, HIV and TB – as they are associated with being gossiped about and shunned. Even participants who were aware of the health risks of being overweight and acknowledged that it was impossible to tell if someone has HIV just from their body size still preferred to gain weight or remain overweight to avoid being ostracised^(106)^.

*‘She would not be happy [to lose weight] because in our community it would be perceived that there is something wrong with her. She would think that the community is looking at her, so she would feel insecure. They would judge her because they say the person is sick and now wants to cover it up by dieting and exercise’ (adult/middle-aged woman, urban, South Africa)* ^(106)^

However, five studies, mainly involving secondary school pupils, reported bullying and exclusion of individuals who were overweight^(98,103,104,112,113)^.

#### Cultural norms, identity and acculturative stress

Two quantitative studies provided evidence for a positive association between exposure to media and a preference for a slimmer body size^(27,28)^. One Egyptian study^(28)^ found that the risk that pictures in female magazines would influence girls’ ideas of a perfect body shape was almost three times higher among female students who were exposed to magazines *v*. not or less frequently exposed. An association between television exposure and female students’ ideas of a perfect body shape was also observed.

If having a large body size was seen as a traditional African ideal or heritage^(42,104,105–108,110,112)^, the media’s promotion of thinness was viewed as the antithesis to this tradition^(104,112,116)^. Participants reported feeling pressure and disappointment from the media’s constant promotion of the thin ideal^(104,112)^.

*‘I hate the media’s emphasis on weight loss because we cannot all be the same. Some people are born thin and others fat. This emphasis makes people think that fat is unacceptable and ugly’* (adolescent girl, urban, South Africa)^(112)^

Some participants wanted to be thin like models because they aspired to a modelling career, or because it was fashionable^(101,104,113)^. Only secondary school pupils reported these views. Black South African participants also reported negative comments from White and Mixed-Ancestry friends/colleagues as influencing their perceptions of body size ideals and causing insecurity in their African heritage beliefs^(98,104)^.

#### Social standing

Body size was viewed as an embodiment of community respectability. Fatness in women pre-marriage was important as it increased their chances of marriage^(109)^. A woman who gained weight soon after marriage was viewed with pride by her in-laws and respected by the community, as her weight gain symbolised being well cared for by her husband and more capable of being fertile and caring for her family^(24,99,100,107,108,110,111,114)^.

*‘For a woman, being overweight suggests that her husband takes good care of her, that he is comfortable, he has money’* (adult woman, urban, Cameroon)^(114)^

A large body size was also associated with general capability, strength, doing well in life^(23,24,42,99–101,103,107,111,113)^ and also represented a symbol of wealth and prosperity^(105,108,110,113,116)^. On the other hand, three studies reported that thinness was associated with affluence^(11,102)^ and strength^(107)^.

## Discussion

### Summary of key findings

The evidence we synthesised on body size preferences from seventy three quantitative, qualitative and mixed methods studies in twenty-one African countries suggests that normal or overweight body sizes are mainly preferred for African females. This echoes earlier findings from authors who conducted research amongst African Americans over 20 years ago^(117)^. This systematic review found evidence that obesity was a preferred body size in only a small number of studies (*n* 4), contrary to widely held assumptions concerning the African region.

We observed differences between rural and urban settings within the countries in terms of body size preferences. Furthermore, we noted that countries with the highest economic development, such as South Africa^(83–85)^, Ghana^(94)^ and Egypt^(28,59,63,82)^, presented several studies showing a preference for thinness, particularly in young women.

Socio-demographic, health-related, psycho-social and socio-cultural factors were found to influence African females’ body size preferences. Factors such as age, SES, place of residence, concern about nutrition-related noncommunicable diseases, clothing and media were found to influence body size preferences in both quantitative and qualitative studies.

Younger participants in Morocco had a higher preference for larger body size whilst in West Africa (Ghana, Gambia, Senegal), East Africa (Seychelles) and Southern Africa (South Africa), they had a greater preference for slimmer body sizes. The effect of age on body size preferences may act via two main pathways. First, lay norms revealed that being overweight was associated with marital and economic status, which could represent proxies for responsibilities associated with age^(102)^. Indeed, age has both a biological and socio-cultural component. In traditional societies, age represents wisdom, responsibility and maturity, especially when associated with moderate overweight for married women^(118)^. Second, young people living in urban areas are more exposed to external media and more likely to question the benefits of overweight^(27,68,69,101)^. Media exposure may influence body size preferences more in adolescent females than in adults. Although older females with a higher BMI tended to desire a slimmer body size, there was some evidence that they become desensitised over time to external media^(119)^, especially as they still valued overweight more, as a symbol of prosperity, fertility and peacefulness in the household^(8,39,97)^.

Both quantitative^(40,48,92)^ and qualitative evidence^(11,102)^ showed that increased SES or better employment was associated with slimmer body size preference. The effect of wealth on body size preferences may act by modulating exposure to factors such as external media, tighter ‘fashionable’ clothing, knowledge of the health risks associated with obesity and better access to food resources in urban environments.

Some themes that emerged from the qualitative synthesis corresponded well with factors reported in the quantitative studies in our review, and in a previously published qualitative review^(120)^. As part of their review, Ozodiegwu and colleagues^(120)^ explored factors in the literature that influence overweight or obesity in women of reproductive age living in four different Sub-Saharan African countries (i.e. Ghana, Kenya, South Africa and Botswana); and therefore, we present some overlapping results. Nevertheless, our mixed-methods review included papers from a broader time range (1985 to August 2019), as well as from twenty-one countries in Africa. In our study, appearance played a role in women’s satisfaction with their body size, as they wanted to look attractive to men and peers. Thinness and fatness were both associated with attractiveness, albeit in different studies. Fatness was associated with having a nice figure; a preference for larger hips was reported by African men and women is in accordance with historical observations from Africa and preferences reported by African Americans^(25,117,120)^. This preference has been explained by evolutionary theory whereby large hips are perceived as indicative of a woman’s suitability for childbearing, and therefore her attractiveness and worth within that society, as historically women’s roles equated to being a wife and mother^(25)^. Thinness, as an embodiment of beauty or looking smart, may highlight women’s changing roles in these societies, or perhaps the encroaching influence of ‘Western’ media and ideals on these traditional views^(22)^. It appears these influences may be more prominent amongst younger participants, as evidenced by the fact that acculturative stress was only reported by younger women. Pressure from peers to look slimmer was also reported among adolescent girls^(120)^. The risk of nutrition-related noncommunicable diseases associated with obesity was found to influence women’s preferred body size. Knowledge of the associated health risks of obesity was not universally reported across studies. Women who were knowledgeable about health were still reluctant to lose weight to avoid the stigma associated with HIV and/or TB, as reported in a previous review^(120)^. This risk of facing ridicule also overshadowed weight loss motivations driven by the prospect of improving personal well-being. This emphasises the importance of community acceptance and social standing on African women’s autonomy. It also highlights how the power of social norms can disempower individuals who might want to manage their weight for personal or health reasons. However, it appears that the HIV and/or TB stigma associated with a phobia for thinness seems to progressively disappear in the most educated young women, while the least educated still persist in valuing stoutness, their socio-cultural environment usually linking thinness with poor healthy status, such as being HIV-positive^(85)^. Another factor that contributed to disempowering women from managing their weight was their understanding of the aetiology of fatness/thinness. The aetiologies cited were generally viewed as being out of the individual’s control and therefore not worth trying to change.

### Strengths and limitations

The main strength of this review is the use of a mixedmethods’ approach to review a large body of evidence. This allowed a holistic understanding of body size preferences for African women and adolescent girls and the factors driving these. Furthermore, all included studies were of medium or high quality. Even if the FRS^(42)^, used in several studies, was initially developed for Caucasian populations, it was subsequently adapted to African populations^(121)^, and a black adolescent female version was also developed and validated^(66)^. These African versions of the FRS were used in some studies included in this review^(73,77,86)^, showing that the FRS has been contextualised to a certain degree to assess body size perceptions of Africans. Whilst the FRS has limitations as a figural scale, its widespread use allows contextual interpretation of the findings across studies. Another strength is that the prevalence of overweight/obesity among the countries included in our study ranged from 21·3 % in Senegal to 72·0 % in Seychelles^(122)^, which can be considered capturing a range of countries at different stages of the nutrition transition in the African continent.

Limitations of the review included generalisability to the African continent, as Africa is a large, economically and ethnically diverse continent. Most of the quantitative studies were conducted in urban settings, limiting their applicability outside urban settings. Studies were conducted in 21/54 African countries and clustered particularly within Southern Africa, West Africa and East Africa, capturing differences between traditional and modern perceptions of female body size. We noted that findings from Morocco appeared quite different from other countries in terms of body size preferences and factors influencing these. Although factors that play a role in influencing body size preferences were identified in the quantitative studies, it was not possible to establish causality, as almost all included studies were cross-sectional.

Studies included in our review were appraised for quality using the QualSyst tool, which assessed both the quality of studies and their reporting. To our knowledge, this was the most appropriate published tool for the range of study designs included in our review. However, we acknowledge the inherent limitations of such tools, which are reliant on what is reported by authors and that are also subjective in nature^(123)^. As such, there is a possibility that study quality may have been misclassified. A specific risk of bias tool may have provided a more objective measure of study quality by focusing on the actual design and conduct of a study. However, no such tool currently exists for the range of study designs included in this review. Furthermore, given the lack of consensus on the issue of excluding studies based on quality^(123)^ and evidence that shows that excluding lower quality studies does not significantly impact on the synthesis of results^(124,125)^, we decided to include all studies regardless of quality in our review as we wanted to capture a fuller account of the topic.

### Policy implications

A preference for overweight body sizes by some African women need not be viewed as a barrier to addressing the rising challenge of overweight and obesity in Africa. A set of interventions, as outlined in the Behaviour Change Wheel^(126)^, could be proposed. For example, emphasis could be placed on education (i.e. increasing knowledge or understanding), persuasion (using communication to stimulate action) and enablement (i.e. increasing means and/or reducing barriers to increase capability or opportunity) interventions. These could include the following actions: (i) providing information to promote a healthy weight through diet and physical activity; (ii) educating the whole community about the gravity of the health risks of obesity and (iii) addressing the underlying attitudes that cause stigmatisation of people with HIV/AIDS and weight loss in general. In addition, conducting mass media campaigns could be used as a vehicle for changing attitudes and risks associated with obesity. Furthermore, younger participants have already expressed unwanted pressure to be slim from the media, as well as some ethnic groups in some African countries; therefore, care must be taken to ensure that educational campaigns do not shift body ideals too far in the other direction and precipitate eating disorders. Qualitative and mixed methods studies focusing on individuals’ perceptions of weight management and education programmes should also be conducted, so that their cultural acceptability and barriers to their use can be determined and used to design culturally acceptable programmes.

## Conclusion

Obesity is an important public health issue with a high prevalence amongst African women. It is generally assumed that larger body sizes are preferred for African females. Overall, the evidence synthesised in this review found a preference for a normal and overweight (not obese) body sizes amongst African females. Factors influencing body size preferences were wide ranging. Whilst middle-aged and elderly women tended to value overweight, young women who are more exposed to norms in ‘Western’ media tended to value thinness and wanted to lose weight to improve health. Nevertheless, these perceived health risks were overshadowed by the community’s negative perceptions towards weight loss and thinness, which are associated with HIV and subsequently negative treatment by the community. A preference for overweight (not obese) body sizes among some African females means that traditional cultural norms may still be an obstacle for preventing overweight/obesity. The findings of this review highlight the need for interventions that account for the array of modifiable factors that maintain these preferences (e.g. education, SES, cultural norms, peer pressure and media) and for interventions to be tailored to different stages of the life course. Emphasis needs to be placed on education to prevent all forms of malnutrition such as promoting a healthy diet to help maintain a healthy body weight and raising awareness on the health consequences of obesity. The widespread preference for normal weight is positive in public health terms; however, the relative valorisation of underweight in adolescents and young women may lead to an increase in body dissatisfaction and eating disorders.

## Supplementary material

For supplementary material accompanying this paper visit https://doi.org/10.1017/S1368980021000768. click here to view supplementary material
