# Supplementary material for: Body size preferences for women and adolescent girls living in Africa: a mixed-methods systematic review
Source: Public Health Nutr. 2021 Feb 17;25(3):738–59. doi: 10.1017/S1368980021000768 (PMC9991778; doi:10.1017/S1368980021000768)
Supplement: Supplementary file 1 [file S1368980021000768sup001.docx]

**Supporting information**

**Supplementary material 1.** Example of search strategy on PsycINFO

| 1. Africa, Western/ or Africa, Northern/ or South Africa/ or Africa, Eastern/ or Africa.mp. or "Africa South of the Sahara"/ or Africa, Central/ or Africa/ or Africa, Southern/  2. Algeria.mp. or Algeria/  3. Angola.mp. or Angola/  4. Benin.mp. or Benin/  5. Botswana.mp. or Botswana/  6. Burkina Faso.mp. or Burkina Faso/  7. Burundi.mp. or Burundi/  8. cabo verde.mp.  9. cape verde.mp. or Cape Verde/  10. cameroon.mp. or Cameroon/  11. central african republic.mp. or Central African Republic/  12. Chad/ or Chad.mp.  13. comoros.mp. or Comoros/  14. congo.mp. or "Democratic Republic of the Congo"/ or Congo/  15. republic of congo.mp.  16. cote d'ivoire.mp. or Cote d'Ivoire/  17. ivory coast.mp. or Cote d'Ivoire/  18. Djibouti.mp. or Djibouti/  19. Egypt.mp. or Egypt/  20. Equatorial Guinea.mp. or Equatorial Guinea/  21. Eritrea.mp. or Eritrea/  22. Ethiopia.mp. or Ethiopia/  23. Gabon.mp. or Gabon/  24. Gambia/ or Gambia.mp.  25. Ghana.mp. or Ghana/  26. Guinea.mp. or Guinea/  27. Guinea-Bissau.mp. or Guinea-Bissau/  28. Kenya.mp. or Kenya/  29. Lesotho.mp. or Lesotho/  30. Liberia/ or Liberia.mp.  31. Libya.mp. or Libya/  32. Madagascar.mp. or Madagascar/  33. Malawi.mp. or Malawi/  34. Mali/ or Mali.mp.  35. Mauritania/ or Mauritania.mp.  36. Mauritius.mp. or Mauritius/  37. Morocco.mp. or Morocco/  38. Mozambique.mp. or Mozambique/  39. Namibia.mp. or Namibia/  40. Niger/ or Niger.mp.  41. Nigeria.mp. or Nigeria/  42. Rwanda.mp. or Rwanda/  43. Sao Tome e principe.mp.  44. (Sao Tome and Principe).mp. [mp=title, abstract, heading word, table of contents, key concepts, original title, tests & measures]  45. Senegal.mp. or Senegal/  46. Seychelles.mp. or Seychelles/  47. Sierra Leone/ or Sierra Leone.mp.  48. Somalia.mp. or Somalia/  49. South Africa.mp. or South Africa/  50. Sudan.mp. or Sudan/  51. Swaziland/ or Swaziland.mp.  52. Tanzania.mp. or Tanzania/  53. Togo.mp. or Togo/  54. Tunisia.mp. or Tunisia/  55. Uganda/ or Uganda.mp.  56. Zambia.mp. or Zambia/  57. Zimbabwe.mp. or Zimbabwe/  58. 2 or 3 or 4 or 5 or 6 or 7 or 8 or 9 or 10 or 11 or 12 or 13 or 14 or 15 or 16 or 17 or 18 or 19 or 20 or 21 or 22 or 23 or 24 or 25 or 26 or 27 or 28 or 29 or 30 or 31 or 32 or 33 or 34 or 35 or 36 or 37 or 38 or 39 or 40 or 41 or 42 or 43 or 44 or 45 or 46 or 47 or 48 or 49 or 50 or 51 or 52 or 53 or 54 or 55 or 56 or 57  59. 1 or 58  60. body size.mp. or *Body Weight/ or *Body Size/  61. body perception.mp. or Self Concept/  62. *body image/ or *weight perception/  63. Body Image/ or Anorexia Nervosa/ or Eating Disorders/ or eating attitudes.mp. or Eating/  64. body esteem.mp.  65. weight control.mp. or Diet, Reducing/  66. body satisfaction.mp.  67. body dissatisfaction.mp.  68. body ideal.mp.  69. weight ideal.mp.  70. size ideal.mp.  71. 60 or 61 or 62 or 63 or 64 or 65 or 66 or 67 or 68 or 69 or 70  72. 59 and 71  73. limit 72 to humans  74. african-american.mp. or African Americans/  75. 72 not 74  76. of.mp. [mp=title, abstract, heading word, table of contents, key concepts, original title, tests & measures]  77. 75 not 76 |
| --- |

## **Supplementary material 2.** Extraction form templates

### *Supplementary material 2a. Quantitative data extraction template*

| **General study information** | | | | | | | | | | | | |
| --- | --- | --- | --- | --- | --- | --- | --- | --- | --- | --- | --- | --- |
| Study ID: | | Extraction Date: | | | | | Reviewer Checking ID: | | | | Study author contact: | |
| Paper Title: | | | | | | | | | | | | |
| Study aim: | | | | | | | | | | | | |
| Notes: | | | | | | | | | | | | |
| **Study Design details** | | | | | | | | | | | | |
| Study design: | | | | | Methodology: | | | | | | | |
| Recruitment: | | | | | | | | | | | | |
| Instrument used: | | | | | How instrument was used: | | | | | | | |
| **Setting** | | | | | | | | | | | | |
| Country: | | | Rural/urban: | | | | | Sample recruited from: | | | | |
| Study start – end date: | | | | | | Corresponding HDI value for year study conducted (development level): | | | | | | |
| **Sample characteristics** | | | | | | | | | | | | |
| Sample size: | Ethnicity: | | | | | Education level: | | Age (range and mean): | | | | SES: |
| Mean (SD) BMI: | % Underweight: | | | | | % Normal weight: | | | | % Overweight: | | % Obese: |
| **Results** | | | | | | | | | | | | |
| Females’ perceptions of their body size mean (SD)/mode: | | | | Females' desired body size mean (SD)/mode: | | | | | FID (difference between perceived and desired): | | | |
| N (%) satisfied with weight/size (% underweight; normal weight; overweight: | | | | N (%) prefer to be larger (% underweight; normal weight; overweight): | | | | | N (%) prefer to be smaller (% underweight; normal weight; overweight): | | | |
| Females' perceptions of size men find most attractive: | | | | Size men find most attractive: | | | | | Difference between females' perceptions of men’s preferences and men's actual preferences: | | | |
| Associations with preference to be larger: | | | | | | | | | | | | |
| Associations with preference to be smaller: | | | | | | | | | | | | |
| Limitations: | | | | | | | | | | | | |
| Conclusions: | | | | | | | | | | | | |
| Notes: | | | | | | | | | | | | |

### *Supplementary material 2b. Qualitative data extraction template*

| **General study information** | | | | | | | | | | | |
| --- | --- | --- | --- | --- | --- | --- | --- | --- | --- | --- | --- |
| Study ID: | | Extraction Date: | | | | Reviewer checking ID: | | | | Study author contact: | |
| Paper Title: | | | | | | | | | | | |
| Study aim: | | | | | | | | | | | |
| Notes: | | | | | | | | | | | |
| **Study Design details** | | | | | | | | | | | |
| Study design: | | | | Methodology: | | | | | | | |
| Recruitment Method: | | | | | | | | | | | |
| Data collection, i.e. focus groups, interviews etc: | | | | Data analysis, i.e. thematic, grounded theory etc: | | | | | | | |
| **Setting** | | | | | | | | | | | |
| Country: | | | Rural/urban: | | | | | Sample recruited from: | | | |
| Study start – end date: | | | | | Corresponding HDI value for year study conducted (development level): | | | | | | |
| **Sample characteristics** | | | | | | | | | | | |
| Sample size: | Ethnicity: | | | | Education level: | | | Age (range and mean): | | | SES: |
| Mean (SD) BMI: | % underweight: | | | | % normal weight: | | | | % overweight: | | % obese: |
| **Results** | | | | | | | | | | | |
| Themes reported: | | | | | | | | | | | |
| **Data extraction framework themes** | | | | | | | | | | | |
| Meanings of fatness: | | | | | | | Corresponding data extracts: | | | | |
| Advantages of being fat: | | | | | | | Corresponding data extracts: | | | | |
| Disadvantages of being fat: | | | | | | | Corresponding data extracts: | | | | |
| Meanings of thinness: | | | | | | | Corresponding data extracts: | | | | |
| Advantages of being thin: | | | | | | | Corresponding data extracts: | | | | |
| Disadvantages of being thin: | | | | | | | Corresponding data extracts: | | | | |
| Attitudes of peers/society towards a fat/thin person: | | | | | | | Corresponding data extracts: | | | | |
| Codes: | | | | | | | | | | | |
| Authors opinions: | | | | | | | | | | | |
| Limitations: | | | | | | | | | | | |
| Conclusions/recommendations: | | | | | | | | | | | |
| Notes: | | | | | | | | | | | |

**Supplementary material 3.** Body image assessment tools

| **Study ID** | **Instrument used** | **Classification** |
| --- | --- | --- |
| Mchiza et al^(77)^ | Figure Rating Scale (FRS) | UW:1-2; NW:3-4; OW:5-6; O:7 and more |
| Matoti-Mvalo & Puoane^(24)^ | Figure Rating Scale (FRS) | UW:1-2; NW:3-4; OW:5-6; O:7 and more |
| Maruf et al^(79)^ | Figure Rating Scale (FRS) | UW:1-2; NW:3-4; OW:5-6; O:7 and more |
| Jackson et al^(63)^ | Figure Rating Scale (FRS) | UW:1-2; NW:3-4; OW:5-6; O:7 and more |
| Okoro & Oyejola^(73)^ | Figure Rating Scale (FRS) | UW:1-2; NW:3-4; OW:5-6; O:7 and more |
| Alwan et al^(75)^ | Figure Rating Scale (FRS) | UW:1-2; NW:3-4; OW:5-6; O:7 and more |
| Benkeser et al^(78)^ | Figure Rating Scale (FRS) | UW:1-2; NW:3-4; OW:5-6; O:7 and more |
| Okoro et al^(86)^ | Figure Rating Scale (FRS) | UW:1-2; NW:3-4; OW:5-6; O:7 and more |
| Ford et al^(59)^ | Figure Rating Scale (FRS) | UW:1-2; NW:3-4; OW:5-6; O:7 and more |
| Szabo & Allwood^(69)^ | Figure Rating Scale (FRS) | UW:1-2; NW:3-4; OW:5-6; O:7 and more |
| Gitau et al^(83)^ | Figure Rating Scale (FRS) | UW:1-2; NW:3-4; OW:5-6; O:7 and more |
| Gitau et al^(84)^ | Figure Rating Scale (FRS) | UW:1-2; NW:3-4; OW:5-6; O:7 and more |
| Gitau et al^(85)^ | Figure Rating Scale (FRS) | UW:1-2; NW:3-4; OW:5-6; O:7 and more |
| Puoane et al^(113)^ | Figure Rating Scale (FRS) | UW:1-2; NW:3-4; OW:5-6; O:7 and more |
| Cohen et al^(97)^ | Figure Rating Scale (FRS) | UW:1-2; NW:3-4; OW:5-6; O:7 and more |
| Amenyah & Michels^(88)^ | Figure Rating Scale (FRS) | UW:1-2; NW:3-4; OW:5-6; O:7 and more |
| Naigaga et al^(96)^ | Figure Rating Scale (FRS) | UW:1-2; NW:3-4; OW:5-6; O:7 and more |
| Croffut et al^(115)^ | Figure Rating Scale (FRS) | UW:1-2; NW:3-4; OW:5-6; O:7 and more |
| Gradidge et al^(87)^ | Figure Rating Scale (FRS) | UW:1-2; NW:3-4; OW:5-6; O:7 and more |
| Prioreschi et al^(95)^ | Figure Rating Scale (FRS) | UW:1-2; NW:3-4; OW:5-6; O:7 and more |
| Michels & Amenyah^(94)^ | Figure Rating Scale (FRS) | UW:1-2; NW:3-4; OW:5-6; O:7 and more |
| Pedro et al^(91)^ | Figure Rating Scale (FRS) | UW:1-2; NW:3-4; OW:5-6; O:7 and more |
| Frederick et al^(71)^ | Contour Drawing Rating Scale (CDRS) | UW:1-3; NW:4-6; OW:7-8; O:9 |
| Swami et al^(27)^ | Contour Drawing Rating Scale (CDRS) | UW:1-3; NW:4-6; OW:7-8; O:9 |
| Gualdi-Russo et al^(90)^ | Contour Drawing Rating Scale (CDRS) | UW:1-3; NW:4-6; OW:7-8; O:9 |
| Ettarh et al^(51)^ | Body image assessment for obesity (BIA-O) | UW:1-5; NW:6-9; OW:10-13; O:14-18 |
| Siervo et al^(68)^ | Body image assessment for obesity (BIA-O) | UW:1-5; NW:6-9; OW:10-13; O:14-18 |
| Furnham & Baguma^(61)^ | Ideal Body Subscale (IBS) | UW:1-4; NW:5-6; OW:7-8; O:9 and more |
| Cogan et al^(52)^ | Ideal Body Subscale (IBS) | UW:1-4; NW:5-6; OW:7-8; O:9 and more |
| Jumah & Duda^(70)^ | Figural Stimuli (FS) | UW:1-4; NW:5-6; OW:7-8; O:9 and more |
| Duda et al^(48)^ | Figural Stimuli (FS) | UW:1-4; NW:5-6; OW:7-8; O:9 and more |
| Rguibi & Belahsen^(8)^ | Body Size Silhouettes (BSS) | UW:1-2; NW:3-4; OW:5-6; O:7 and more |
| Caradas et al^(53)^ | Body Silhouette Chart (BSC) | UW:1-2; NW:3-4; OW:5-6; O:7-8 |
| Yepes et al^(92)^ | Body Image Instrument (BII) | UW:1-2; NW:3-4; OW:5-6; O:7 and more |
| Holdsworth et al^(40)^ | Silhouette Photographs (SP) | NW:1-2; OW:3; O:4-6 |
| Swami et al^(41)^ | Photographic Figure Rating Scale (PFRS) | UW:1-4; NW:5-6; OW:7-8; O:9-10 |
| Cohen et al^(42)^ | Body Image Scale (BIS) | UW:1; NW:2; OW:3; O:4-6 |
| Cohen et al^(114)^ | Body Size Scale (BSS) | UW:1; NW:2-4; OW:5-6; O:7 and more |
| Cohen et al^(116)^ | Body Size Scale (BSS) | UW:1; NW:2-4; OW:5-6; O:7 and more |
| Macia et al^(93)^ | Body Size Scale (BSS) | UW:1; NW:2-4; OW:5-6; O:7 and more |
| Toriola et al^(62)^ | Questionnaire item | Desired BMI asked |

Supplementary material 4. Quality appraisal scores

*Supplementary material 4a. Quality appraisal scores for quantitative studies*

| **Study ID** | **Quality Appraisal for quantitative studies** | | | | | | | | | | | | | |
| --- | --- | --- | --- | --- | --- | --- | --- | --- | --- | --- | --- | --- | --- | --- |
|  | Q1. Question/objective | Q2. Study design | Q3. Subject selection | Q4. Subject characteristics | Q5. Random allocation | Q6. Investigator blinding | Q7. Subject blinding | Q8. Outcome measure | Q9. Sample size | Q10. Data analysis | Q11. Estimate of variance | Q12. Control for confounding | Q13. Result reporting | Q14. Conclusions |
| Salokun & Toriola^(58)^ | 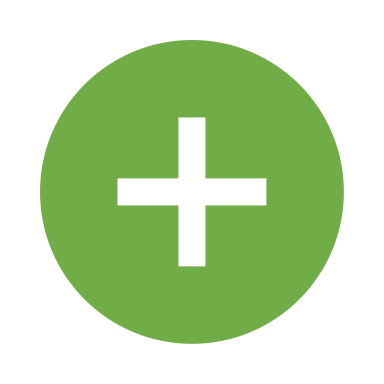 | 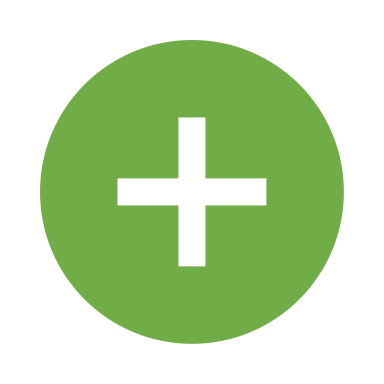 | 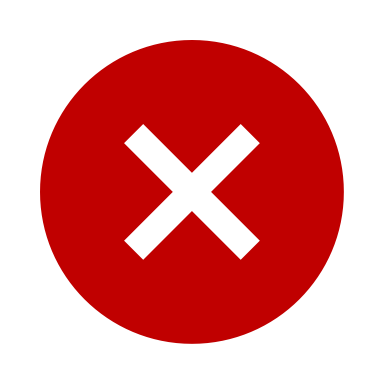 | 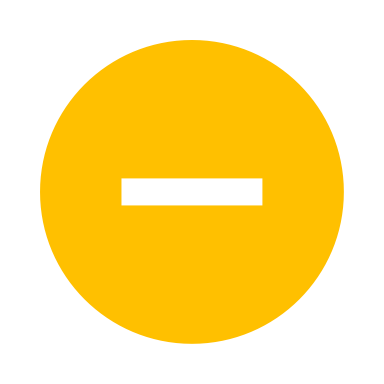 | n/a | n/a | n/a | 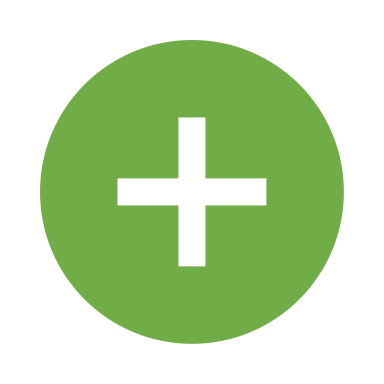 | 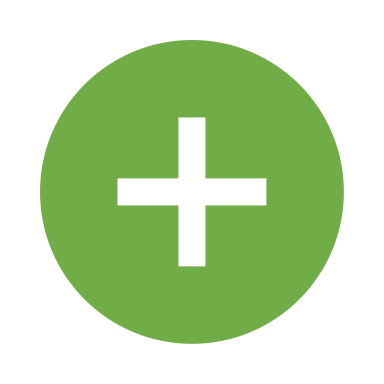 | 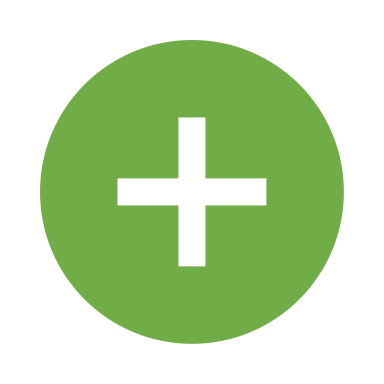 | 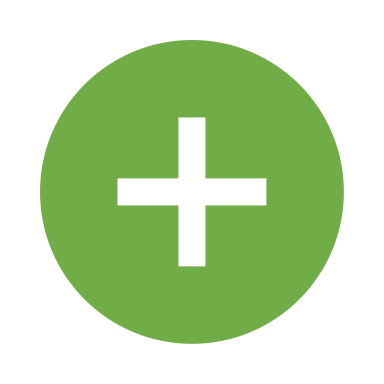 | 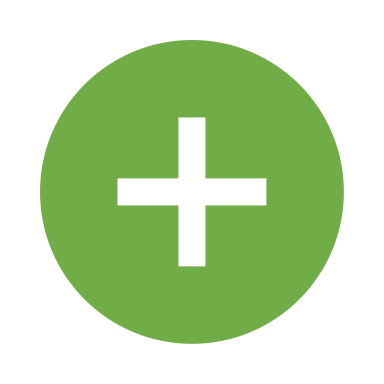 | 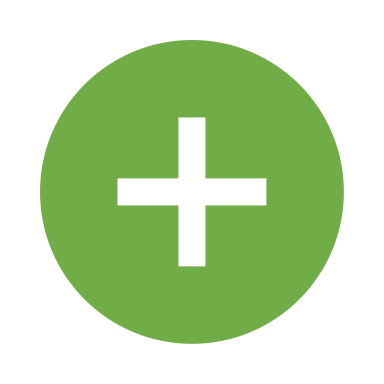 | 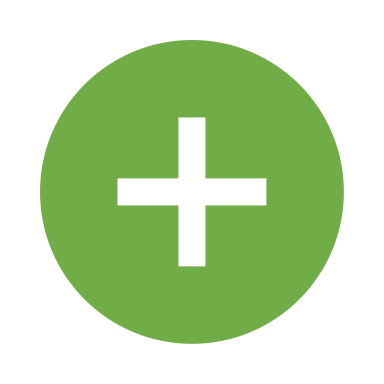 |
| Ford et al^(59)^ | 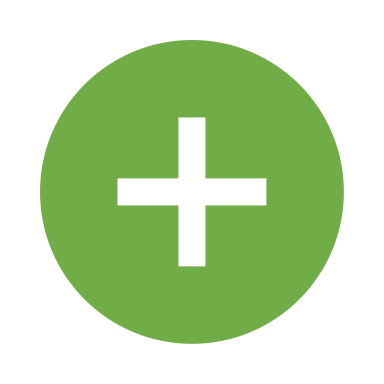 | 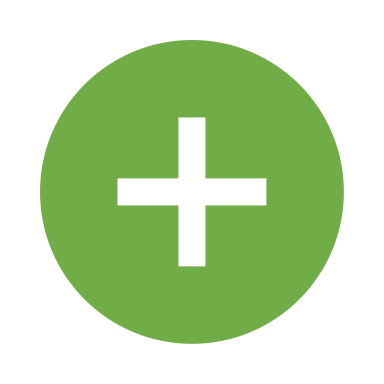 | 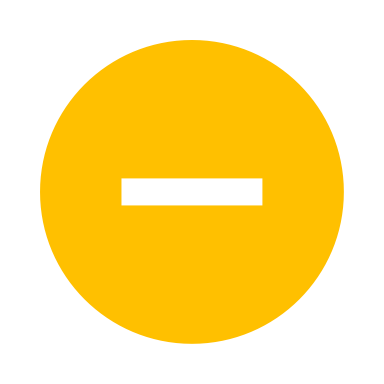 | 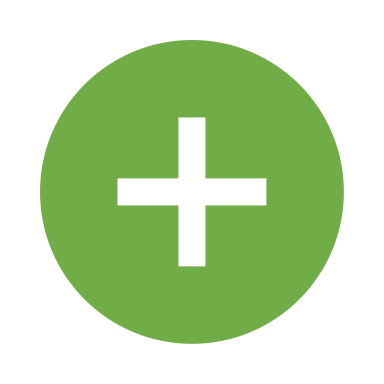 | n/a | n/a | n/a | 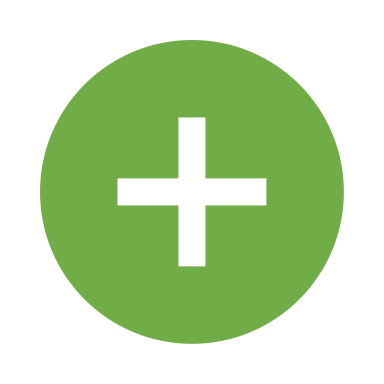 | 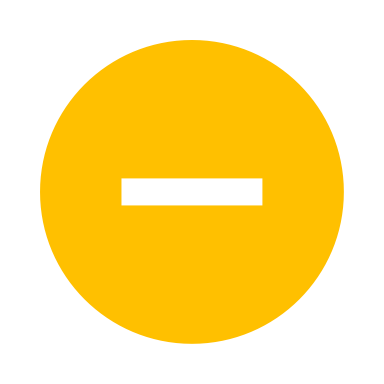 | 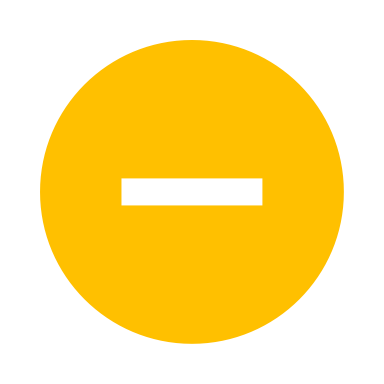 | 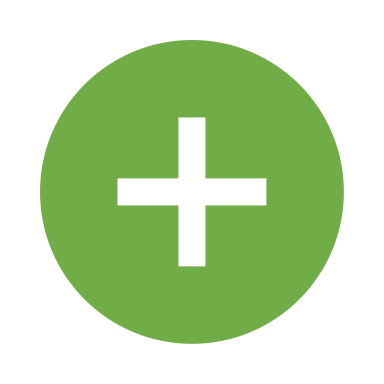 | 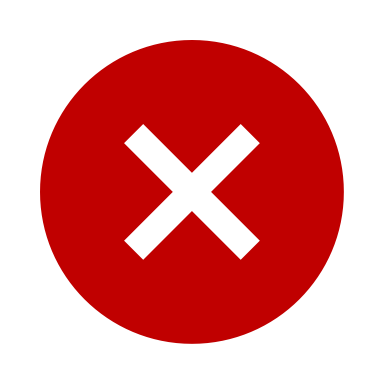 | 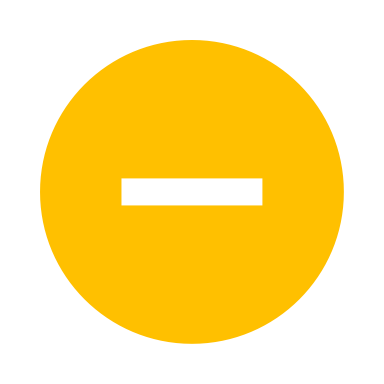 | 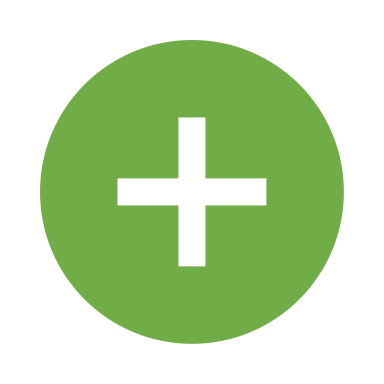 |
| Walker et al^(60)^ | 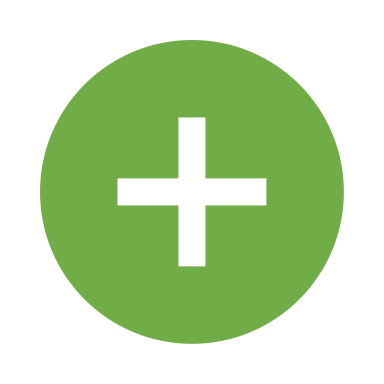 | 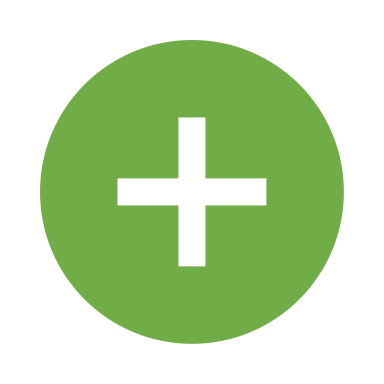 | 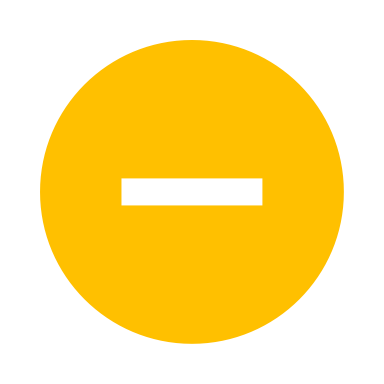 | 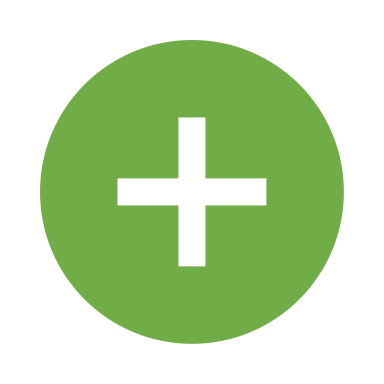 | n/a | n/a | n/a | 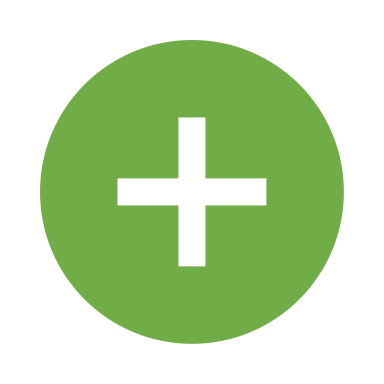 | 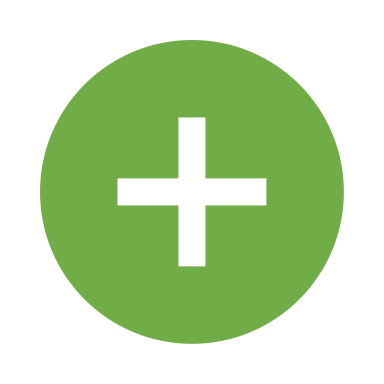 | 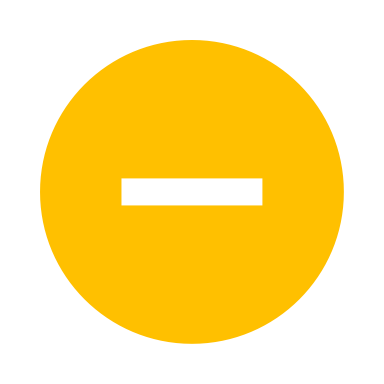 | 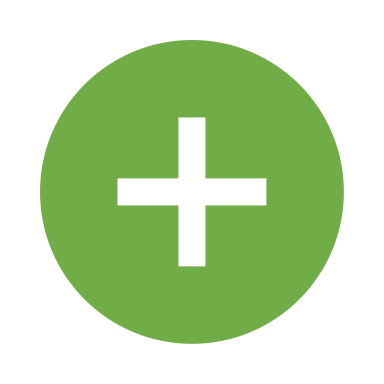 | 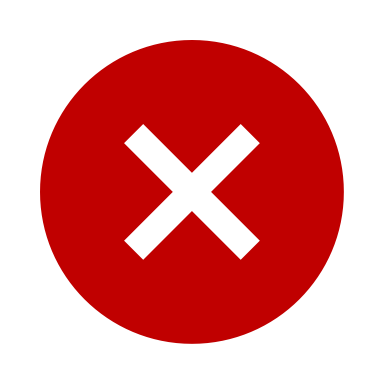 | 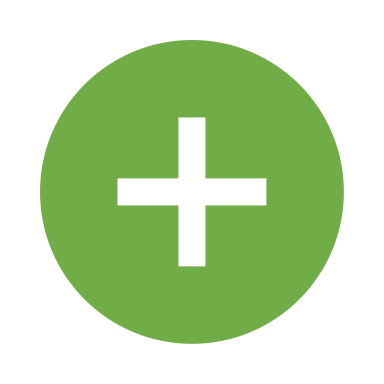 | 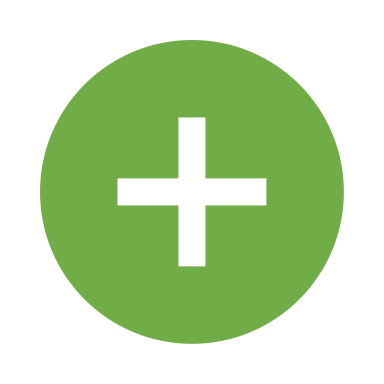 |
| Furnham & Baguma^(61)^ | 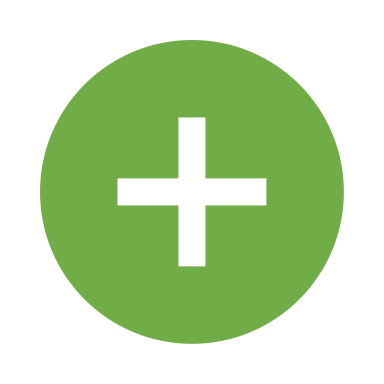 | 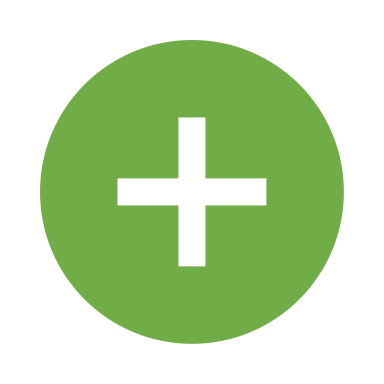 | 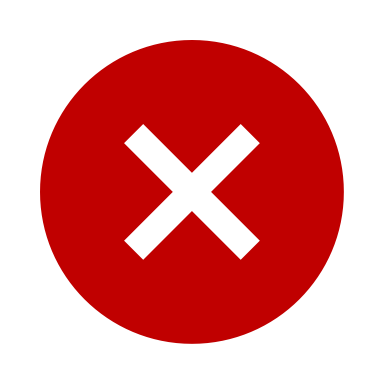 | 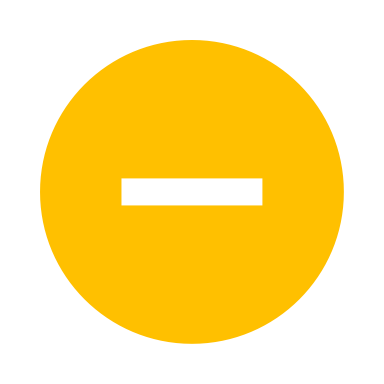 | n/a | n/a | n/a | 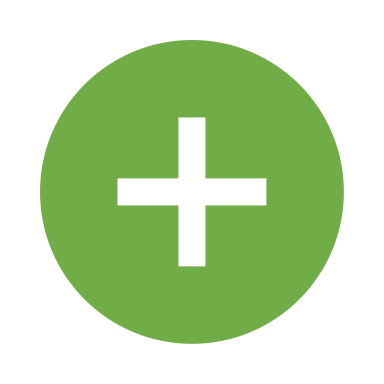 | 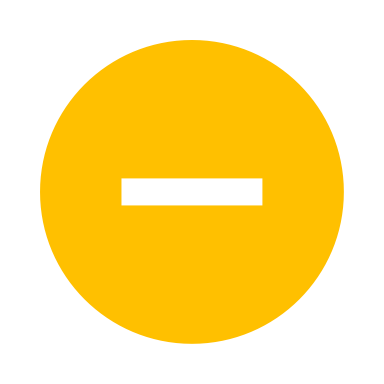 | 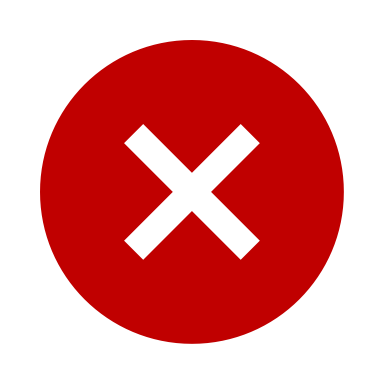 | 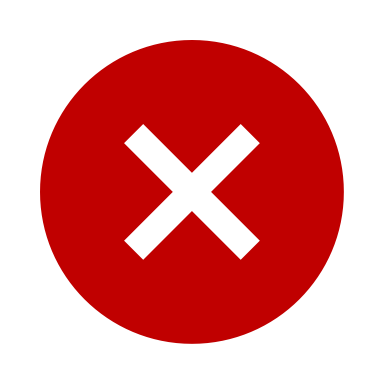 | 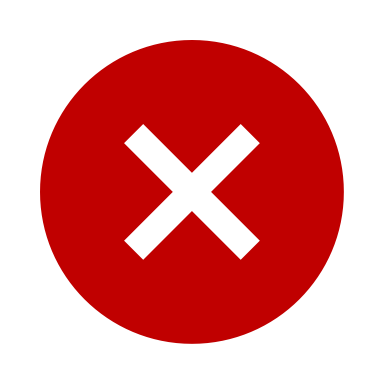 | 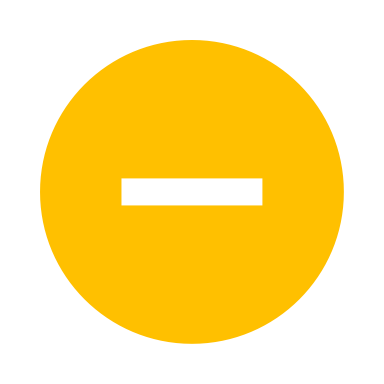 | 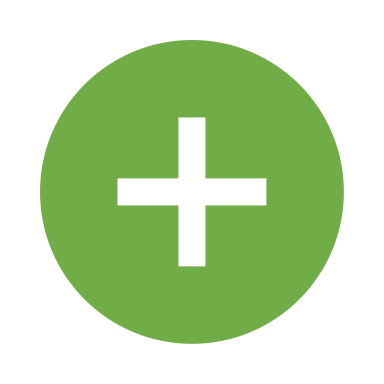 |
| Cogan et al^(52)^ | 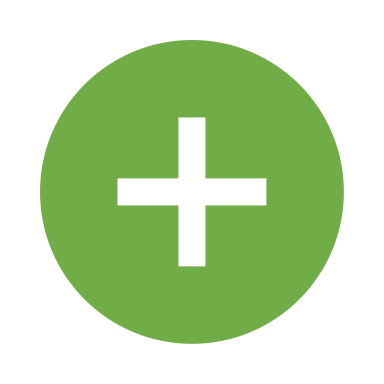 | 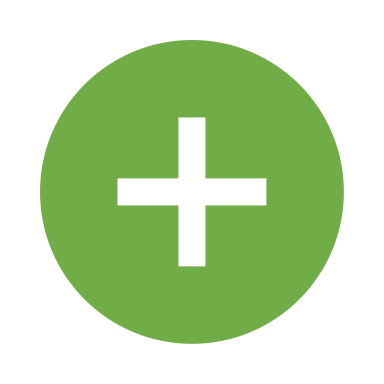 | 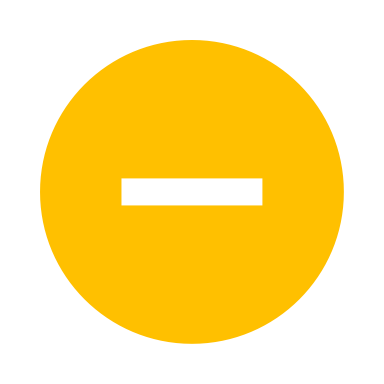 | 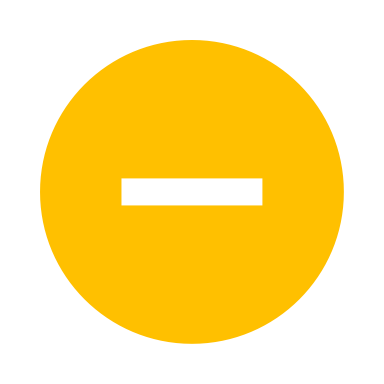 | n/a | n/a | n/a | 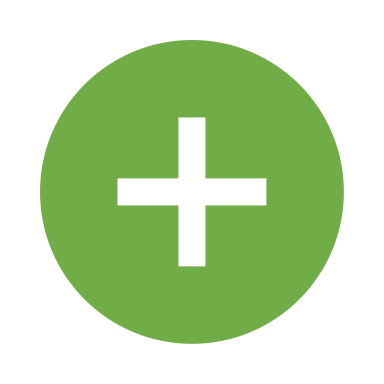 | 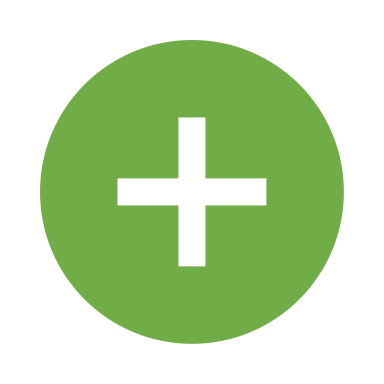 | 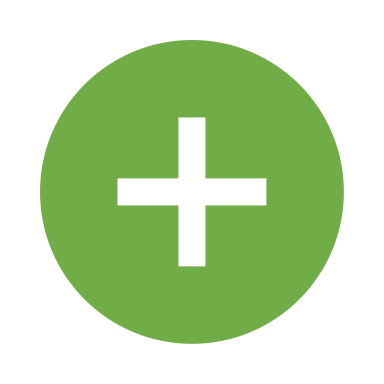 | 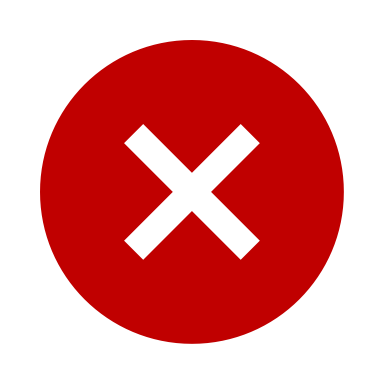 | 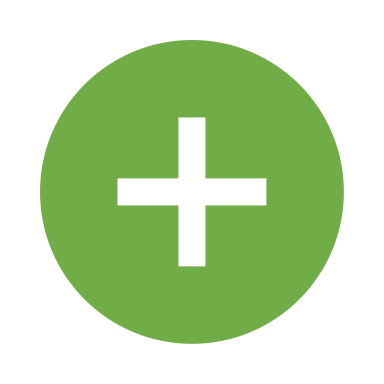 | 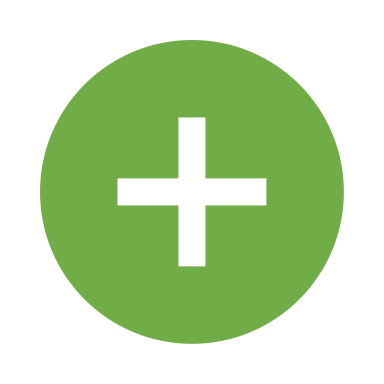 | 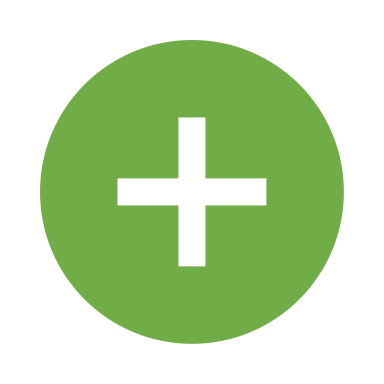 |
| Toriola et al^(62)^ | 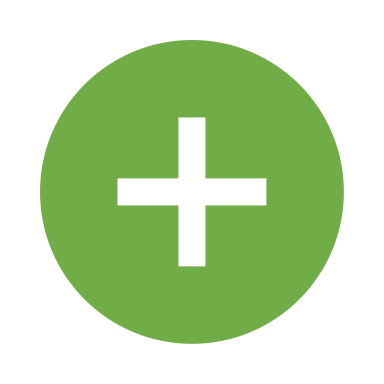 | 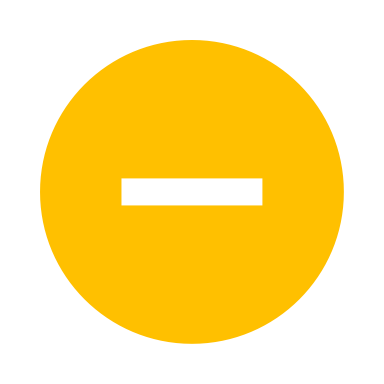 | 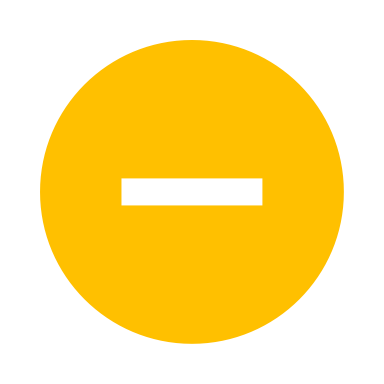 | 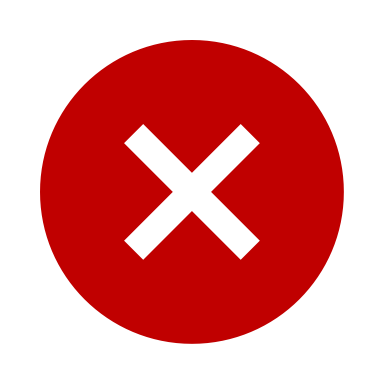 | n/a | n/a | n/a | 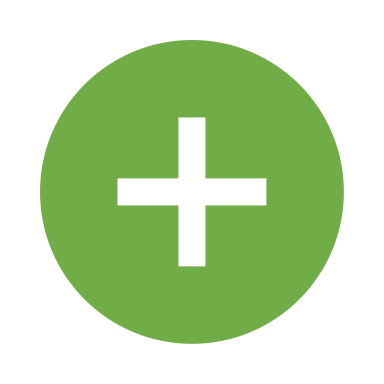 | 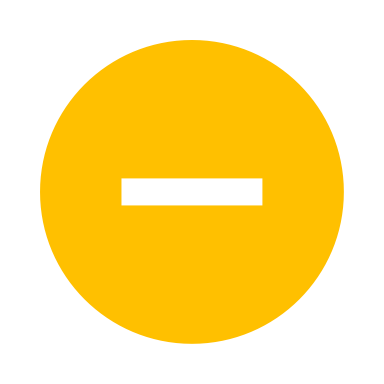 | 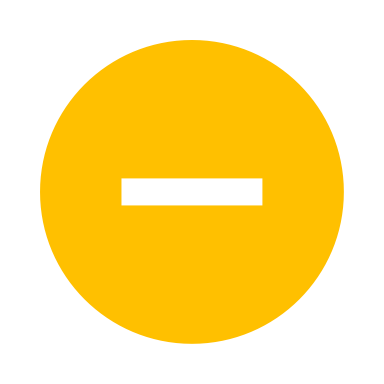 | 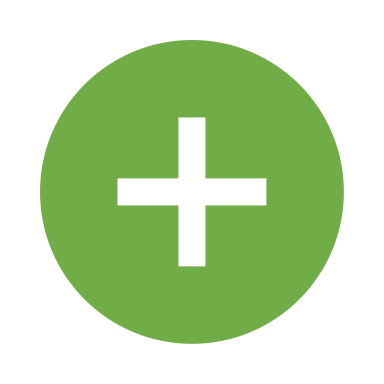 | 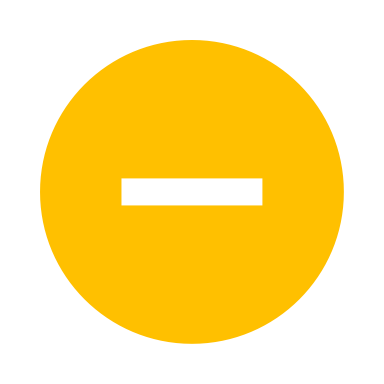 | 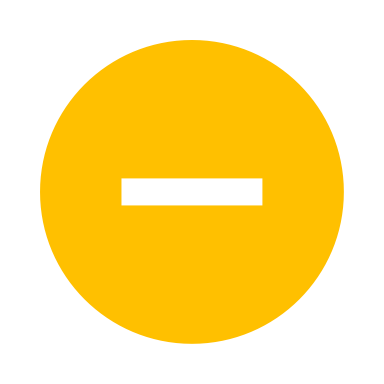 | 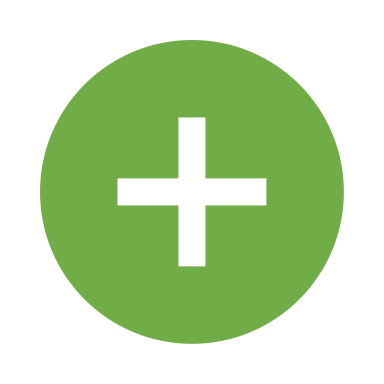 |
| Caradas et al^(53)^ | 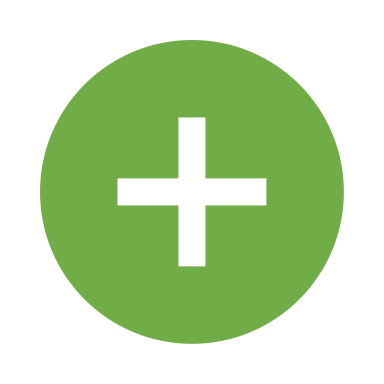 | 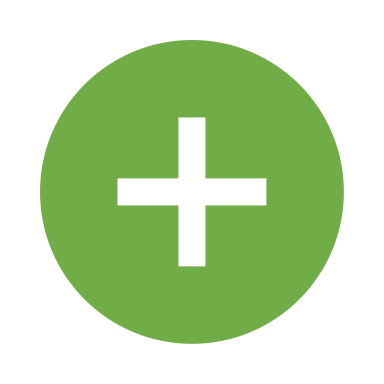 | 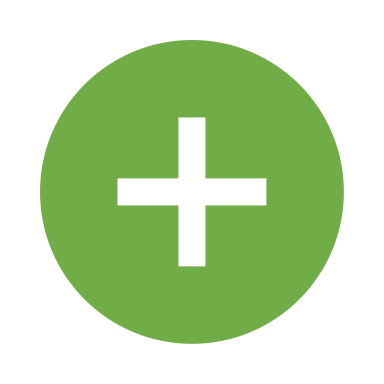 | 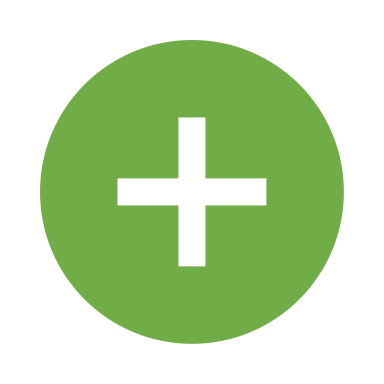 | n/a | n/a | n/a | 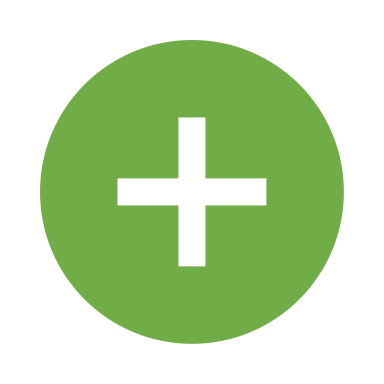 | 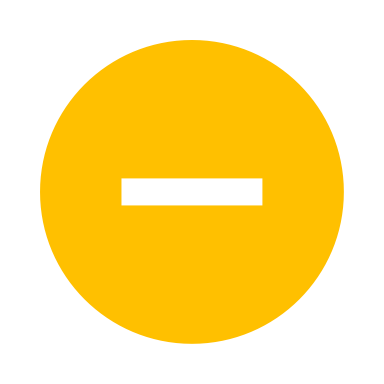 | 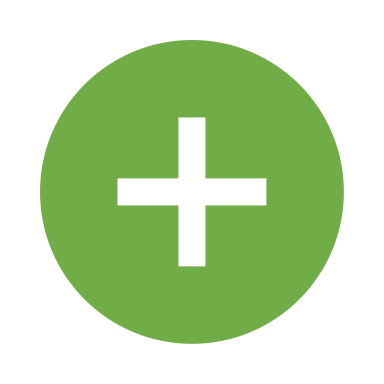 | 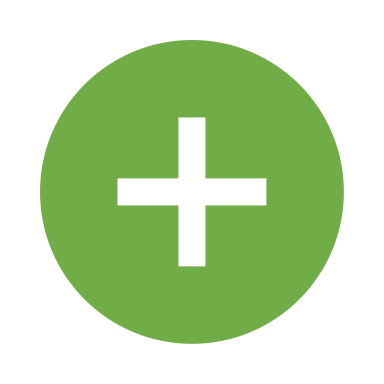 | 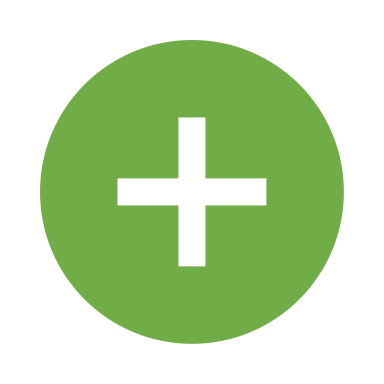 | 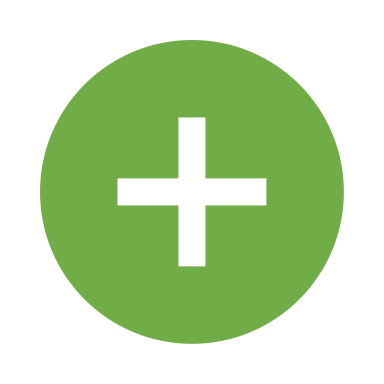 | 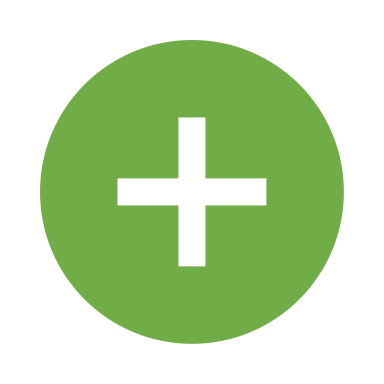 |
| Jackson et al^(63)^ | 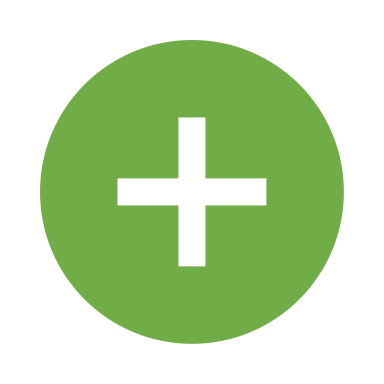 | 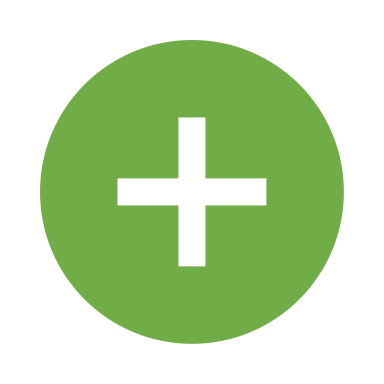 | 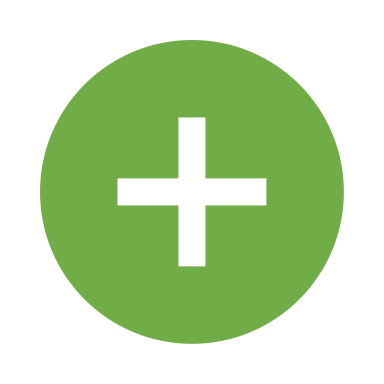 | 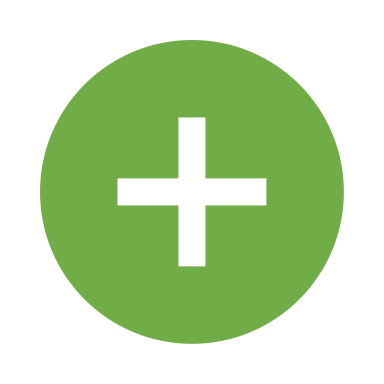 | n/a | n/a | n/a | 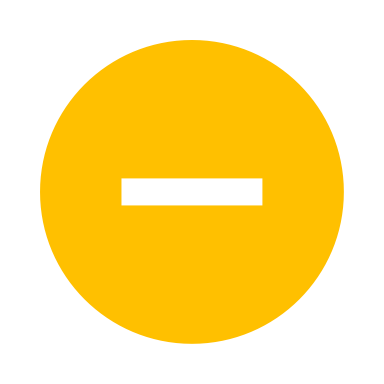 | 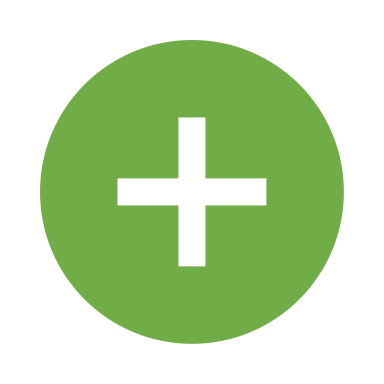 | 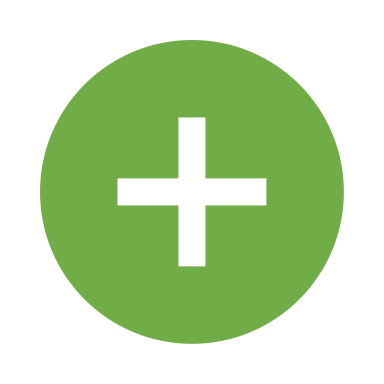 | 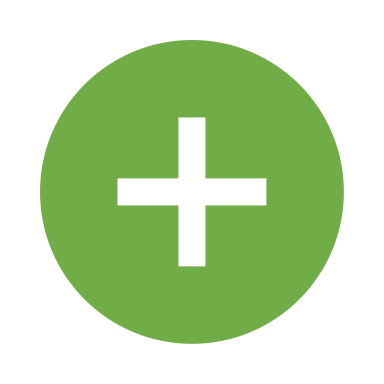 | 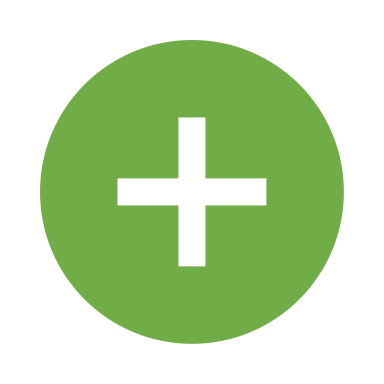 | 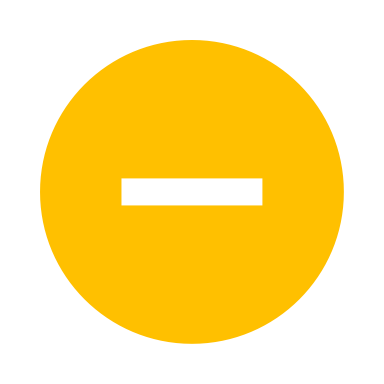 | 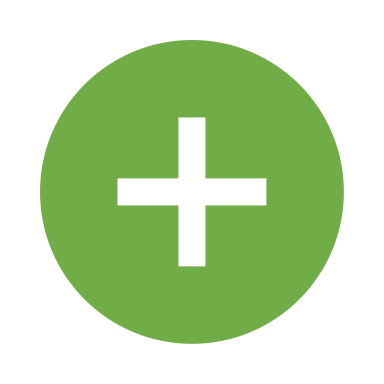 |
| Holdsworth et al^(40)^ | 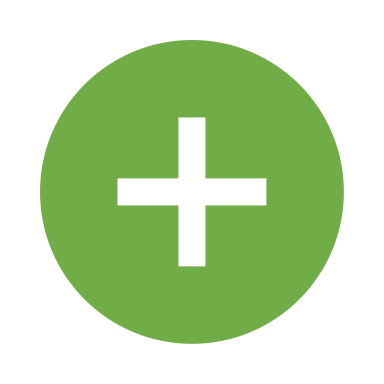 | 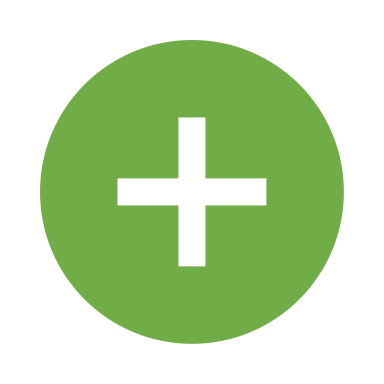 | 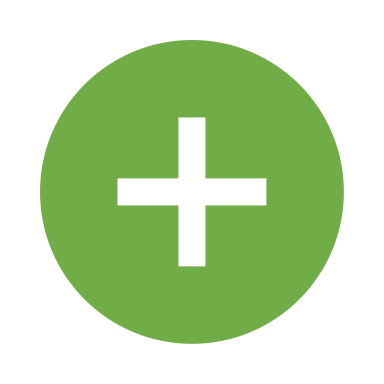 | 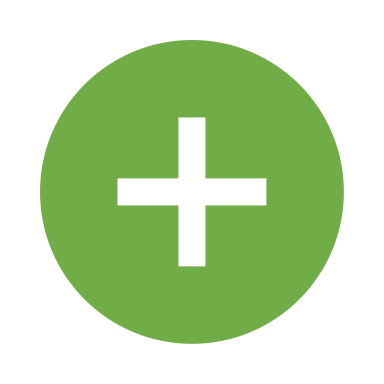 | n/a | n/a | n/a | 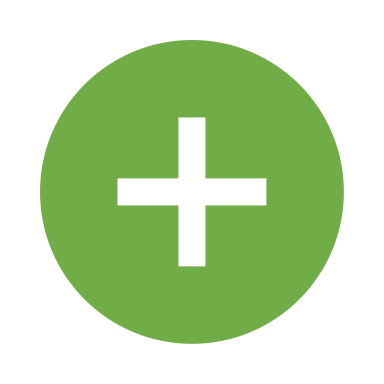 | 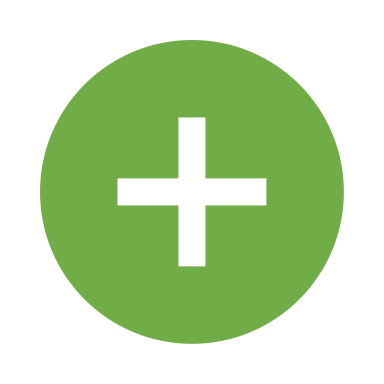 | 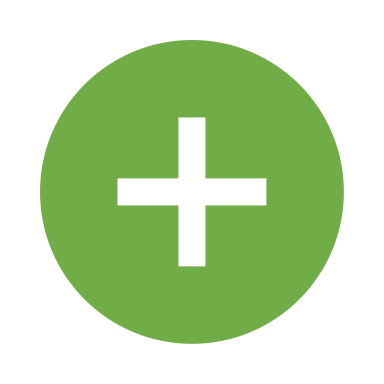 | 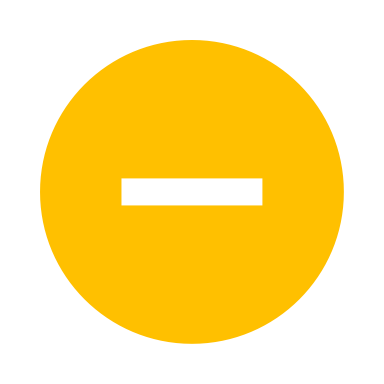 | 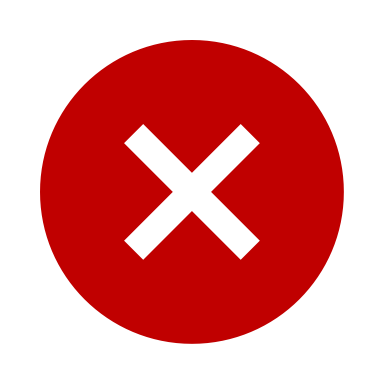 | 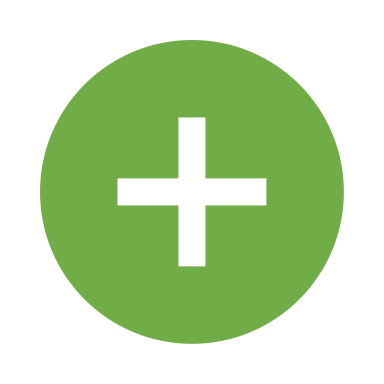 | 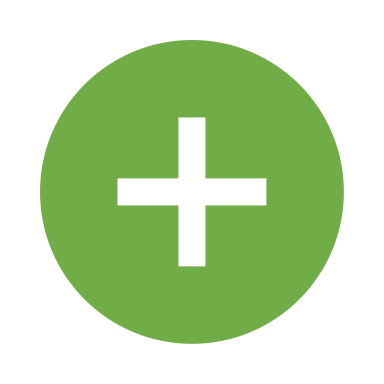 |
| Singh^(64)^ | 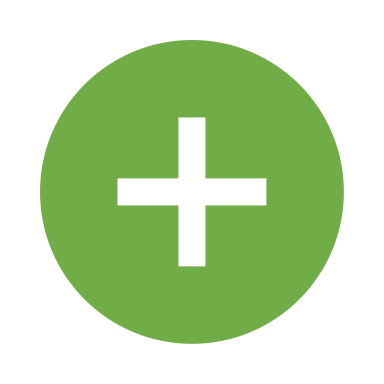 |  |  |  | n/a | n/a | n/a |  |  |  |  |  |  |  |
| Faber & Kruger^(65)^ |  |  |  |  | n/a | n/a | n/a |  |  |  |  |  |  |  |
| Mciza et al^(66)^ |  |  |  |  | n/a | n/a | n/a |  |  |  |  |  |  |  |
| Puoane et al^(111)^ |  |  |  |  | n/a | n/a | n/a |  |  |  |  |  |  |  |
| Duda et al^(67)^ |  |  |  |  | n/a | n/a | n/a |  |  |  |  |  |  |  |
| Rguibi & Belahsen^(8)^ |  |  |  |  | n/a | n/a | n/a |  |  |  |  |  |  |  |
| Rguibi & Belahsen^(9)^ |  |  |  |  | n/a | n/a | n/a |  |  |  |  |  |  |  |
| Siervo et al^(68)^ |  |  |  |  | n/a | n/a | n/a |  |  |  |  |  |  |  |
| Szabo & Allwood^(69)^ |  |  |  |  | n/a | n/a | n/a |  |  |  |  |  |  |  |
| Duda et al^(48)^ |  |  |  |  | n/a | n/a | n/a |  |  |  |  |  |  |  |
| Jumah & Duda^(70)^ |  |  |  |  | n/a | n/a | n/a |  |  |  |  |  |  |  |
| Bodiba et al^(112)^ |  |  |  |  | n/a | n/a | n/a |  |  |  |  |  |  |  |
| Frederick et al^(71)^ |  |  |  |  | n/a | n/a | n/a |  |  |  |  |  |  |  |
| Lahmam et al^(72)^ |  |  |  |  | n/a | n/a | n/a |  |  |  |  |  |  |  |
| Okoro & Oyejola^(73)^ |  |  |  |  | n/a | n/a | n/a |  |  |  |  |  |  |  |
| Mwaba & Roman^(74)^ |  |  |  |  | n/a | n/a | n/a |  |  |  |  |  |  |  |
| Puoane et al^(113)^ |  |  |  |  | n/a | n/a | n/a |  |  |  |  |  |  |  |
| Swami et al^(27)^ |  |  |  |  | n/a | n/a | n/a |  |  |  |  |  |  |  |
| Alwan et al^(75)^ |  |  |  |  | n/a | n/a | n/a |  |  |  |  |  |  |  |
| Coetzee & Perrett^(76)^ |  |  |  |  | n/a | n/a | n/a |  |  |  |  |  |  |  |
| Matoti-Mvalo & Puoane^(24)^ |  |  |  |  | n/a | n/a | n/a |  |  |  |  |  |  |  |
| McHiza et al^(77)^ |  |  |  |  | n/a | n/a | n/a |  |  |  |  |  |  |  |
| Benkeser et al^(78)^ |  |  |  |  | n/a | n/a | n/a |  |  |  |  |  |  |  |
| Maruf et al^(79)^ |  |  |  |  | n/a | n/a | n/a |  |  |  |  |  |  |  |
| Swami et al^(41)^ |  |  |  |  | n/a | n/a | n/a |  |  |  |  |  |  |  |
| Bhurtun & Jeewon^(80)^ |  |  |  |  | n/a | n/a | n/a |  |  |  |  |  |  |  |
| Cohen et al^(42)^ |  |  |  |  | n/a | n/a | n/a |  |  |  |  |  |  |  |

NO i.e. not reported or addressed; PARTIAL i.e. partially reported/addressed; YES i.e. fully reported/addressed; n/a - not applicable to study

*Supplementary material 4a. Quality appraisal scores for quantitative studies (continued)*

| **Study ID** | **Quality Appraisal for quantitative studies** | | | | | | | | | | | | | |
| --- | --- | --- | --- | --- | --- | --- | --- | --- | --- | --- | --- | --- | --- | --- |
|  | Q1. Question/objective | Q2. Study design | Q3. Subject selection | Q4. Subject characteristics | Q5. Random allocation | Q6. Investigator blinding | Q7. Subject blinding | Q8. Outcome measure | Q9. Sample size | Q10. Data analysis | Q11. Estimate of variance | Q12. Control for confounding | Q13. Result reporting | Q14. Conclusions |
| Ettarh et al^(51)^ |  |  |  |  | n/a | n/a | n/a |  |  |  |  |  |  |  |
| Jafri et al^(81)^ |  |  |  |  | n/a | n/a | n/a |  |  |  |  |  |  |  |
| ElAnsari et al^(82)^ |  |  |  |  | n/a | n/a | n/a |  |  |  |  |  |  |  |
| Gitau et al^(85)^ |  |  |  |  | n/a | n/a | n/a |  |  |  |  |  |  |  |
| Gitau et al^(83)^ |  |  |  |  | n/a | n/a | n/a |  |  |  |  |  |  |  |
| Gitau et al^(84)^ |  |  |  |  | n/a | n/a | n/a |  |  |  |  |  |  |  |
| Musaiger & Mannai^(28)^ |  |  |  |  | n/a | n/a | n/a |  |  |  |  |  |  |  |
| Okoro et al^(73)^ |  |  |  |  | n/a | n/a | n/a |  |  |  |  |  |  |  |
| Gradidge et al^(87)^ |  |  |  |  | n/a | n/a | n/a |  |  |  |  |  |  |  |
| Amenyah & Michels^(88)^ |  |  |  |  | n/a | n/a | n/a |  |  |  |  |  |  |  |
| Caleyachetty et al^(89)^ |  |  |  |  | n/a | n/a | n/a |  |  |  |  |  |  |  |
| Gualdi-Russo et al^(90)^ |  |  |  |  | n/a | n/a | n/a |  |  |  |  |  |  |  |
| Pedro et al^(91)^ |  |  |  |  | n/a | n/a | n/a |  |  |  |  |  |  |  |
| Yepes et al^(92)^ |  |  |  |  | n/a | n/a | n/a |  |  |  |  |  |  |  |
| Cohen et al^(114)^ |  |  |  |  | n/a | n/a | n/a |  |  |  |  |  |  |  |
| Macia et al^(93)^ |  |  |  |  | n/a | n/a | n/a |  |  |  |  |  |  |  |
| Michels & Amenyah^(94)^ |  |  |  |  | n/a | n/a | n/a |  |  |  |  |  |  |  |
| Prioreschi et al^(95)^ |  |  |  |  | n/a | n/a | n/a |  |  |  |  |  |  |  |
| Croffut et al^(115)^ |  |  |  |  | n/a | n/a | n/a |  |  |  |  |  |  |  |
| Naigaga et al^(96)^ |  |  |  |  | n/a | n/a | n/a |  |  |  |  |  |  |  |
| Cohen et al^(97)^ |  |  |  |  | n/a | n/a | n/a |  |  |  |  |  |  |  |
| Cohen et al^(116)^ |  |  |  |  | n/a | n/a | n/a |  |  |  |  |  |  |  |

NO i.e. not reported or addressed; PARTIAL i.e. partially reported/addressed; YES i.e. fully reported/addressed; n/a - not applicable to study

*Supplementary material 4b. Quality appraisal scores for qualitative studies*

| **Study ID** | **Checklist for assessing the quality of qualitative studies** | | | | | | | | | |
| --- | --- | --- | --- | --- | --- | --- | --- | --- | --- | --- |
|  | Q1. Question/objective | Q2. Study design | Q3. Context | Q4. Theoretical framework | Q5. Sampling | Q6. Data collection | Q7. Data analysis | Q8. Verification procedure | Q9. Conclusions | Q10. Reflexivity |
| Mvo et al^(98)^ |  |  |  |  |  |  |  |  |  |  |
| Treloar et al^(99)^ |  |  |  |  |  |  |  |  |  |  |
| Puoane et al^(111)^ |  |  |  |  |  |  |  |  |  |  |
| Kiawi et al^(100)^ |  |  |  |  |  |  |  |  |  |  |
| Dapi et al^(101)^ |  |  |  |  |  |  |  |  |  |  |
| Batnitzky^(102)^ |  |  |  |  |  |  |  |  |  |  |
| Bodiba et al^(112)^ |  |  |  |  |  |  |  |  |  |  |
| Ezekiel et al^(23)^ |  |  |  |  |  |  |  |  |  |  |
| Puoane et al^(113)^ |  |  |  |  |  |  |  |  |  |  |
| Batnitzky^(11)^ |  |  |  |  |  |  |  |  |  |  |
| Matoti-Mvalo & Puoane^(24)^ |  |  |  |  |  |  |  |  |  |  |
| Shaibu et al^(103)^ |  |  |  |  |  |  |  |  |  |  |
| Cohen et al^(42)^ |  |  |  |  |  |  |  |  |  |  |
| Morris & Szabo^(104)^ |  |  |  |  |  |  |  |  |  |  |
| Ahmed & Saltus^(105)^ |  |  |  |  |  |  |  |  |  |  |
| Draper et al^(106)^ |  |  |  |  |  |  |  |  |  |  |
| Pradeilles^(107)^ |  |  |  |  |  |  |  |  |  |  |
| Okop et al^(108)^ |  |  |  |  |  |  |  |  |  |  |
| Phillips et al^(109)^ |  |  |  |  |  |  |  |  |  |  |
| Cohen et al^(114)^ |  |  |  |  |  |  |  |  |  |  |
| Croffut et al^(115)^ |  |  |  |  |  |  |  |  |  |  |
| Cohen et al^(116)^ |  |  |  |  |  |  |  |  |  |  |
| Tateyama et al^(110)^ |  |  |  |  |  |  |  |  |  |  |

NO i.e. not reported or addressed; PARTIAL i.e. partially reported/addressed; YES i.e. fully reported/addressed; n/a - not applicable to study

**Supplementary material 5.** Dimensions of body size preferences and/or drivers of these preferences in the included studies

| **Study ID** | **Dimension 1   Ideal body size  (n=46)** | **Dimension 2 Perceived body size**  **(n=19)** | **Dimension 3  Body satisfaction  (n=44)** | **Quantitative drivers of body size ideals  (n=29)** | **Qualitative drivers of body size ideals   (n=23)** |
| --- | --- | --- | --- | --- | --- |
| **Quantitative articles** | | | | | |
| Salokun & Toriola^(58)^ | ✓ | ✕ | ✕ | ✕ | ✕ |
| Ford et al^(59)^ | ✓ | ✕ | ✓ | ✕ | ✕ |
| Walker et al^(60)^ | ✕ | ✕ | ✓ | ✕ | ✕ |
| Furnham & Baguma^(61)^ | ✓ | ✕ | ✕ | ✕ | ✕ |
| Cogan et al^(52)^ | ✓ | ✕ | ✓ | ✕ | ✕ |
| Toriola et al^(62)^ | ✕ | ✕ | ✓ | ✕ | ✕ |
| Caradas et al^(53)^ | ✓ | ✓ | ✓ | ✕ | ✕ |
| Jackson et al^(63)^ | ✓ | ✓ | ✓ | ✓ | ✕ |
| Holdsworth et al^(40)^ | ✓ | ✕ | ✓ | ✓ | ✕ |
| Singh^(64)^ | ✓ | ✕ | ✕ | ✕ | ✕ |
| Faber & Kruger^(65)^ | ✓ | ✕ | ✓ | ✓ | ✕ |
| Mciza et al^(66)^ | ✓ | ✓ | ✓ | ✕ | ✕ |
| Duda et al^(67)^ | ✕ | ✕ | ✕ | ✓ | ✕ |
| Rguibi & Belahsen^(8)^ | ✓ | ✕ | ✓ | ✓ | ✕ |
| Rguibi & Belahsen^(9)^ | ✕ | ✕ | ✓ | ✕ | ✕ |
| Siervo et al^(68)^ | ✓ | ✕ | ✓ | ✓ | ✕ |
| Szabo & Allwood^(69)^ | ✓ | ✕ | ✓ | ✓ | ✕ |
| Duda et al^(48)^ | ✓ | ✓ | ✓ | ✓ | ✕ |
| Jumah & Duda^(70)^ | ✓ | ✓ | ✓ | ✓ | ✕ |
| Frederick et al^(71)^ | ✓ | ✕ | ✓ | ✓ | ✕ |
| Lahmam et al^(72)^ | ✕ | ✓ | ✓ | ✓ | ✕ |
| Okoro & Oyejola^(73)^ | ✓ | ✕ | ✓ | ✕ | ✕ |
| Mwaba & Roman^(74)^ | ✕ | ✕ | ✓ | ✕ | ✕ |
| Swami et al^(27)^ | ✓ | ✕ | ✓ | ✓ | ✕ |
| Alwan et al^(75)^ | ✓ | ✓ | ✓ | ✓ | ✕ |
| Coetzee & Perrett^(76)^ | ✕ | ✕ | ✕ | ✕ | ✕ |
| Mchiza et al^(77)^ | ✓ | ✕ | ✓ | ✕ | ✕ |
| Benkeser et al^(78)^ | ✓ | ✓ | ✓ | ✓ | ✕ |
| Maruf et al^(79)^ | ✓ | ✓ | ✓ | ✓ | ✕ |
| Swami et al^(41)^ | ✓ | ✕ | ✓ | ✕ | ✕ |
| Bhurtun & Jeewon^(80)^ | ✕ | ✓ | ✓ | ✓ | ✕ |
| Ettarh et al^(51)^ | ✓ | ✓ | ✓ | ✕ | ✕ |
| Jafri et al^(81)^ | ✕ | ✕ | ✕ | ✓ | ✕ |
| ElAnsari et al^(82)^ | ✕ | ✕ | ✕ | ✕ | ✕ |
| Gitau et al^(85)^ | ✓ | ✕ | ✓ | ✓ | ✕ |
| Gitau et al^(83)^ | ✓ | ✕ | ✓ | ✕ | ✕ |
| Gitau et al^(84)^ | ✓ | ✕ | ✕ | ✕ | ✕ |
| Musaiger & Mannai^(28)^ | ✓ | ✕ | ✕ | ✓ | ✕ |
| Okoro et al^(73)^ | ✓ | ✕ | ✓ | ✓ | ✕ |

**Supplementary material 5.** Dimensions of body size preferences and/or drivers of these preferences in the included studies (continued)

| **Study ID** | **Dimension 1   Ideal body size  (n=46)** | **Dimension 2 Perceived body size**  **(n=19)** | **Dimension 3  Body satisfaction  (n=44)** | **Quantitative drivers of body size ideals  (n=29)** | **Qualitative drivers of body size ideals   (n=23)** |
| --- | --- | --- | --- | --- | --- |
| **Quantitative articles** | | | | | |
| Gradidge et al^(87)^ | ✓ | ✓ | ✓ | ✓ | ✕ |
| Amenyah & Michels^(88)^ | ✓ | ✕ | ✓ | ✓ | ✕ |
| Caleyachetty et al^(89)^ | ✕ | ✕ | ✕ | ✕ | ✕ |
| Gualdi-Russo et al^(90)^ | ✓ | ✓ | ✓ | ✕ | ✕ |
| Pedro et al^(91)^ | ✓ | ✕ | ✓ | ✓ | ✕ |
| Yepes et al^(92)^ | ✓ | ✕ | ✕ | ✓ | ✕ |
| Macia et al^(93)^ | ✓ | ✕ | ✓ | ✕ | ✕ |
| Michels & Amenyah^(94)^ | ✓ | ✕ | ✓ | ✕ | ✕ |
| Prioreschi et al^(95)^ | ✓ | ✕ | ✓ | ✓ | ✕ |
| Naigaga et al^(96)^ | ✓ | ✓ | ✓ | ✕ | ✕ |
| Cohen et al^(97)^ | ✓ | ✓ | ✓ | ✓ | ✕ |
| **Qualitative articles** | | | | | |
| Mvo et al^(98)^ | ✕ | ✕ | ✕ | ✕ | ✓ |
| Treloar et al^(99)^ | ✕ | ✕ | ✕ | ✕ | ✓ |
| Kiawi et al^(100)^ | ✕ | ✕ | ✕ | ✕ | ✓ |
| Dapi et al^(101)^ | ✕ | ✕ | ✕ | ✕ | ✓ |
| Batnitzky^(102)^ | ✕ | ✕ | ✕ | ✕ | ✓ |
| Ezekiel et al^(23)^ | ✕ | ✕ | ✕ | ✕ | ✓ |
| Batnitzky^(11)^ | ✕ | ✕ | ✕ | ✕ | ✓ |
| Shaibu et al^(103)^ | ✕ | ✕ | ✕ | ✕ | ✓ |
| Morris & Szabo^(104)^ | ✕ | ✕ | ✕ | ✕ | ✓ |
| Ahmed & Saltus^(105)^ | ✕ | ✕ | ✕ | ✕ | ✓ |
| Draper et al^(106)^ | ✕ | ✕ | ✕ | ✕ | ✓ |
| Pradeilles^(107)^ | ✕ | ✕ | ✕ | ✕ | ✓ |
| Okop et al^(108)^ | ✕ | ✕ | ✕ | ✕ | ✓ |
| Phillips et al^(109)^ | ✕ | ✕ | ✕ | ✕ | ✓ |
| Tateyama et al^(110)^ | ✕ | ✕ | ✕ | ✕ | ✓ |
| **Mixed Methods articles** | | | | | |
| Puoane et al^(111)^ | ✓ | ✓ | ✓ | ✕ | ✓ |
| Bodiba et al^(112)^ | ✕ | ✕ | ✕ | ✕ | ✓ |
| Puoane et al^(113)^ | ✓ | ✕ | ✕ | ✕ | ✓ |
| Matoti-Mvalo & Puoane^(24)^ | ✓ | ✓ | ✕ | ✕ | ✓ |
| Cohen et al^(42)^ | ✓ | ✓ | ✓ | ✓ | ✓ |
| Cohen et al^(114)^ | ✓ | ✕ | ✓ | ✕ | ✓ |
| Croffut et al^(115)^ | ✓ | ✓ | ✓ | ✓ | ✓ |
| Cohen et al^(116)^ | ✓ | ✕ | ✓ | ✓ | ✓ |

Note: There were three studies that matched our inclusion criteria and were hence included in the review but could not be used for the synthesis (Caleyachetty et al^89^; Coetzee & Perrett^76^; ElAnsari et al^82^)
